# Elastocaloric determination of the phase diagram of Sr_2_RuO_4_

**DOI:** 10.1038/s41586-022-04820-z

**Published:** 2022-07-13

**Authors:** You-Sheng Li, Markus Garst, Jörg Schmalian, Sayak Ghosh, Naoki Kikugawa, Dmitry A. Sokolov, Clifford W. Hicks, Fabian Jerzembeck, Matthias S. Ikeda, Zhenhai Hu, B. J. Ramshaw, Andreas W. Rost, Michael Nicklas, Andrew P. Mackenzie

**Affiliations:** 1 Max Planck Institute for Chemical Physics of Solids, Dresden, Germany; 2 Institut für Theoretische Festkörperphysik, Karlsruher Institut für Technologie, Karlsruhe, Germany; 3 Institut für QuantenMaterialien und Technologien, Karlsruher Institut für Technologie, Karlsruhe, Germany; 4 Institut für Theorie der Kondensierten Materie, Karlsruher Institut für Technologie, Karlsruhe, Germany; 5 Laboratory of Atomic and Solid State Physics, Cornell University, Ithaca, NY USA; 6 National Institute for Materials Science, Tsukuba, Japan; 7 School of Physics and Astronomy, University of Birmingham, Birmingham, UK; 8 Geballe Laboratory for Advanced Materials, Stanford University, Stanford, CA USA; 9 Department of Applied Physics, Stanford University, Stanford, CA USA; 10 Stanford Institute for Materials and Energy Sciences, SLAC National Accelerator Laboratory, Menlo Park, CA USA; 11 Scottish Universities Physics Alliance, School of Physics and Astronomy, University of St Andrews, St Andrews, UK; 12 Max Planck Institute for Solid State Research, Stuttgart, Germany

**Keywords:** Superconducting properties and materials, Phase transitions and critical phenomena

## Abstract

One of the main developments in unconventional superconductivity in the past two decades has been the discovery that most unconventional superconductors form phase diagrams that also contain other strongly correlated states. Many systems of interest are therefore close to more than one instability, and tuning between the resultant ordered phases is the subject of intense research^1^. In recent years, uniaxial pressure applied using piezoelectric-based devices has been shown to be a particularly versatile new method of tuning^2,3^, leading to experiments that have advanced our understanding of the fascinating unconventional superconductor Sr_2_RuO_4_ (refs. ^4–9^). Here we map out its phase diagram using high-precision measurements of the elastocaloric effect in what we believe to be the first such study including both the normal and the superconducting states. We observe a strong entropy quench on entering the superconducting state, in excellent agreement with a model calculation for pairing at the Van Hove point, and obtain a quantitative estimate of the entropy change associated with entry to a magnetic state that is observed in proximity to the superconductivity. The phase diagram is intriguing both for its similarity to those seen in other families of unconventional superconductors and for extra features unique, so far, to Sr_2_RuO_4_.

## Abstract (web summary)

The phase diagram of the unconventional superconductor Sr_2_RuO_4_ in both normal and superconducting states is mapped out using high-precision measurements of the elastocaloric effect, showing similarities to other unconventional superconductors as well as unique features.

## Main

To establish the phase diagram of an unconventional superconductor, it is necessary to have both an effective means of tuning it and methods to investigate the resultant changes to its physical properties. In most of the systems studied so far, tuning methods such as chemical composition, magnetic field, electric field, hydrostatic and epitaxial pressure have been used. Each has its advantages and drawbacks, which ultimately determine the methods used to study the resultant phases and their interplay. An ideal method of study is one that has sensitivity to several phases simultaneously, and in particular to their boundaries. In magnetically tuned systems, the magnetocaloric effect has proved to be of particular utility. Under adiabatic conditions, the rate of change of the sample temperature with applied magnetic field *H* provides direct information on the heat capacity *C*_H_ and entropy *S*, through the well-known relationship

1 $${\left.\frac{\Delta T}{\Delta H}\right|}_{S}\cong -\frac{T}{{C}_{{\rm{H}}}}{\left.\frac{\partial S}{\partial H}\right|}_{T},$$

which has been used to good effect to establish the *H*–*T* phase diagrams of, for example, URu_2_Si_2_ (ref. ^10^) and Sr_3_Ru_2_O_7_ (ref. ^11^). As a tuning parameter, magnetic field brings advantages in terms of directionality and the ability to change symmetry, but also has the clear disadvantage that sufficiently high fields usually destroy, rather than promote, superconductivity. Uniaxial pressure brings the same advantage in terms of ‘selective symmetry breaking’ and does not automatically compete with superconductivity. In systems with a strong elastic response, the elastocaloric effect is a direct analogue of the magnetocaloric effect:

2 $${\left.\frac{\Delta T}{\Delta \varepsilon }\right|}_{S}\cong -\frac{T}{{C}_{\varepsilon }}{\left.\frac{\partial S}{\partial \varepsilon }\right|}_{T}$$

in which *C*_*ε*_ is the specific heat at constant strain *ε* and ${\left.\frac{\partial S}{\partial \varepsilon }\right|}_{T}$ is the strain derivative of the entropy at constant temperature. In the special case of an isotropic volume strain Δ*ε* = Δ*V/V*, then $-\frac{1}{T}\frac{\Delta T}{\Delta \varepsilon }$ measures the famous Grüneisen parameter Γ originally introduced in 1908 (ref. ^12^) and extensively studied in, for example, heavy fermion materials^13^, but a generalized version of the Grüneisen parameter can also be defined for any combination of strain tensor components. If the relevant strain tunes the material through a quantum phase transition, the appropriate generalized Grüneisen parameter is an excellent tool with which to classify that transition^14,15^.

Although used widely in association with materials with large elastic responses, to the extent that it has been proposed for cooling technologies^16,17^, direct measurement of the elastocaloric effect has been much less widely used in the field of unconventional superconductivity or correlated electron physics, partly because the expected signal size is much smaller. Here we build on recent work using a.c. methods to perform high-resolution measurements of Δ*T*/Δ*ε* in Fe-based superconductors^18–20^ to study the elastocaloric effect in Sr_2_RuO_4_. As described in detail in Methods, we superimpose a small oscillatory component on the background steady strain and lock into the oscillatory component of the thermal response, which directly measures Δ*T*/Δ*ε*. We achieve the extremely high temperature measurement precision of approximately 2 μK (√Hz)^−1^ and use it to map out the phase diagram between 1 K and 8 K, for applied compressive strains along the [100] crystal axis of up to *ε*_100_ = −0.7%, performing checks to ensure that we are close to the adiabatic limit for which Equation (2) applies. Our data allow us to determine Γ_100_, the Grüneisen parameter for uniaxial stress applied along [100].

**Fig. 1:**
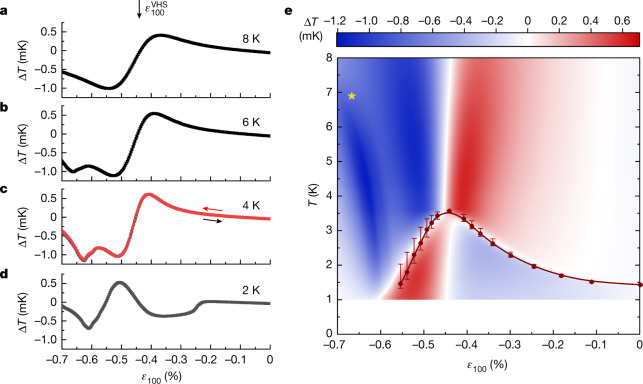
Response of the elastocaloric effect as a function of strain. **a**–**d**, The magnitude of the measured a.c. temperature against strain *ε*_100_ at different average sample temperatures, measured at 1,513 Hz and an excitation amplitude ${\varepsilon }_{100}^{{\rm{exc}}}$ between 2.9 × 10^−6^ and 3.5 × 10^−6^. The strain at which the Van Hove singularity is traversed ${\varepsilon }_{100}^{{\rm{VHS}}}=-\,0\,.\,44 \%$ is indicated in panel **a**. The sign change of Δ*T* at ${\varepsilon }_{100}^{{\rm{VHS}}}$ corresponds to a maximum in the entropy. In panel **c**, data are shown for downsweeps and upsweeps at a rate of approximately 1% per hour. **e**, Colour map of the elastocaloric effect. Notice the pronounced entropy quench at *T*_c_, at which the entropy changes from being maximal at ${\varepsilon }_{100}^{{\rm{VHS}}}$ for *T* > *T*_c_ to forming a minimum at *T* < *T*_c_. The solid red circles are the superconducting transition temperatures determined from measurements of the heat capacity^30^. The yellow star indicates the magnetic phase transition temperature obtained from μSR data^23^, which agrees with the phase boundary identified by the dark blue contrast seen for *ε*_100_ between −0.6% and −0.7% in the elastocaloric effect. See Extended Data Figs. 1, 2 and 3 for further data.

Sample raw data for isothermal strain sweeps at 8 K, 6 K, 4 K and 2 K are shown in Fig. 1a–d. Much can be learned from a qualitative inspection of the results. At 8 K, the data are seen to show the profile expected for a system in which a peak in entropy is studied under quasi-adiabatic conditions: the derivative changes sign at *ε*_100_ ≅ −0.44%, in line with previous estimates for the strain at which a Van Hove singularity is traversed at a so-called Lifshitz transition^5,8,21,22^.

When the temperature has been lowered to 6 K, the signal at the Van Hove strain ${\varepsilon }_{100}^{{\rm{VHS}}}$ remains similar, but a pronounced extra dip is seen in the signal at around *ε*_100_ ≈ −0.6%. By 4 K, this dip has moved to slightly lower absolute strain and becomes stronger. The signal at 2 K looks similar to those at 4 K and 6 K at high strain but is very different in the region between −0.2% and −0.6% strain. Instead of a maximum of entropy at the Van Hove strain, there is now a minimum, along with a sharp step in the elastocaloric signal at *ε*_100_ ≅ −0.23%. Remarkably, this large change in the entropic properties is the result of the onset of superconductivity, as demonstrated by constructing the empirical phase diagram shown in Fig. 1e from interpolating the results of strain sweeps from 71 different temperatures, as described in Methods.

The high resolution of our experiments allows the straightforward identification of several key features from inspection of the raw data in Fig. 1e. First, above *T*_c_, the strain at which the elastocaloric signal changes sign is nearly independent of temperature. This is the intuitive expectation for the elastocaloric signal of traversing a Van Hove singularity, which is expected to be independent of temperature in this temperature range because it is set by an underlying feature in the band structure and therefore determined by much higher energy scales. Within experimental uncertainty, it coincides with the maximum value of *T*_c_ and the strain at which the Van Hove singularity is observed to be crossed in photoemission experiments^8^. Second, the dispersion with strain of the dip seen for *ε*_100_ values of less than −0.6% (Fig. 1b–d) is reminiscent of that of a phase boundary. Third, the entropic signal of entering the superconducting state is extremely pronounced. The maximum in entropy as a function of strain at the Van Hove singularity is quenched, turning into a minimum below *T*_c_. Away from the Van Hove point, the elastocaloric effect changes sign and almost reverses its magnitude near *T*_c_. No signature of a second transition within the superconducting state^23^ is resolved.

**Fig. 2:**
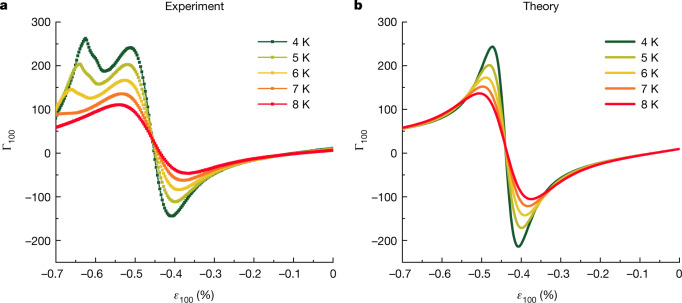
Grüneisen scaling. **a**, Experimental data converted to absolute units using a temperature-independent and strain-independent scale factor determined at 6 K using the procedure described in Methods. **b**, Theoretical calculations of Γ_100_ in the temperature range *T* ≥ 4 K using a simple single-band, two-dimensional model. The sign change at *ε*_100_ ≈ −0.44% can clearly be attributed to crossing the Van Hove point. A Grüneisen scaling collapse is observed for small strains *ε*_100_ > −0.2% in both panels. The extra peak at large strains for *ε*_100_ around −0.65% in panel **a** is attributed to magnetism that is not captured by the theory of panel **b**. A more realistic model including the full three-dimensional dispersion of Sr_2_RuO_4_ (Supplementary Information) gives essentially the same theoretical results.

To frame a more in-depth analysis of our data, we turn to the behaviour of the relevant Grüneisen parameter ${\Gamma }_{100}\equiv -\frac{1}{T}\frac{\Delta T}{\Delta {\varepsilon }_{100}}$, converting the raw data to absolute units using the procedure described in Methods. In systems governed by a single energy scale, such as Fermi liquids, Γ is independent of temperature. As a result, Grüneisen scaling is expected with curves at all temperatures collapsing onto each other and deviations from this scaling indicating proximity to critical points or phase transitions^14,15^. We show this scaling in Fig. 2a for temperatures greater than the maximum superconducting transition temperature of 3.5 K. It is seen to be excellent for −0.3% < *ε*_100_ < 0. Between −0.3% and −0.55%, the departure from scaling is of the kind qualitatively expected for proximity to a quantum phase transition, in this case, the Lifshitz transition at *ε*_100_ = −0.44%. For strains between −0.6% and −0.7%, the Grüneisen scaling is also poorly obeyed, supporting the hypothesis that the feature in this region (now a peak rather than a dip because of the sign convention of the Grüneisen parameter) marks a phase transition.

As a complement to the experimental data, we have calculated the expected behaviour of the Grüneisen parameter as a function of [100] strain using a two-dimensional tight-binding model for the relevant *γ* band derived from a combination of de Haas–van Alphen and angle-resolved photoemission experiments on unstrained Sr_2_RuO_4_ (ref. ^24^). Full details are given in Methods and Supplementary Information.

The results are shown in Fig. 2b for the same range of temperatures as those in Fig. 2a. The qualitative agreement at strains $\left|{\varepsilon }_{100}\right| < \left|{\varepsilon }_{100}^{{\rm{VHS}}}\right|$ is distinctive, especially given the simplicity of the model. The shape of the curves, the strain range over which the Grüneisen scaling is obeyed and even the zero crossing near zero strain (a consequence of the initial splitting of the zero-field Van Hove singularity owing to the Poisson effect) are all seen in both experiment and theory. By contrast, below 8 K, the behaviour for strains beyond ${\varepsilon }_{100}^{{\rm{VHS}}}$ is considerably different, emphasizing that the experimental data are picking up a phase transition not predicted by the tight-binding model. In isolation, the elastocaloric data give no microscopic information on the nature of the high-strain phase, but a point established in a recent muon spin relaxation measurement (marked by the yellow star in Fig. 1e) shows that it is magnetic, and probably a finite-*Q* state^23^. Establishing the boundary of this new phase is one of our key findings.

**Fig. 3:**
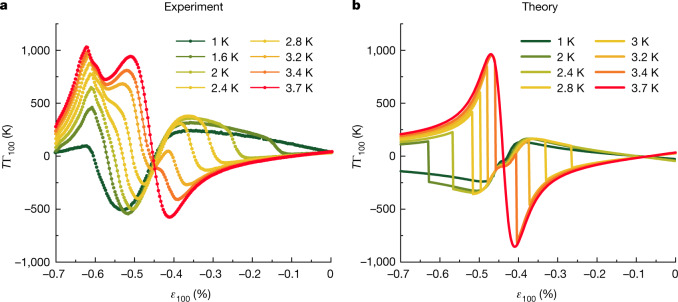
Elastocaloric effect. **a**, Experimental data converted to absolute units using the procedure described in the caption to Fig. 2. **b**, Theoretical calculations with a full gap at the Van Hove point of *T*Γ_100_ in the temperature range 1 K ≤ *T* ≤ 3.7 K using a simple single-band, two-dimensional model. The discontinuities in panel **b** identify the phase transition between the metallic and superconducting phases; these singularities are broadened in the experimental data in panel **a**. The extra peak at large strains for *ε*_100_ around −0.65% is attributed to magnetism.

Next, we turn our attention to lower temperatures. In Fig. 3a, we show elastocaloric data at a range of temperatures between 3.7 K and 1 K. At 3.7 K, the sample is non-superconducting across the entire strain range, whereas at 1 K, it is superconducting for *ε*_100_ between −0.55% and 0. The behaviour at intermediate temperatures is prominent. After following the expected normal state behaviour at low strains, the signal abruptly reverses in sign owing to the entropy quench discussed above. On the high-strain side, the departure from the superconducting state becomes harder to distinguish and the signal increases rapidly in the region where superconducting and magnetic order approach each other.

Because there is, to our knowledge, no precedent in the literature of measurement of the elastocaloric signal on entry to the superconducting state, we constructed an illustrative model to frame the discussion of Fig. 3a. In Fig. 3b, we show the elastocaloric response obtained in a simple calculation: the density of states of the empirically constrained tight-binding model is combined with a strain-independent and *k*-independent pairing potential *V* to calculate the transition temperature of a hypothetical weak-coupled Bardeen–Cooper–Schrieffer superconductor that is fully gapped at the Van Hove points (see Supplementary Information). We do not claim that this model gives a full description of the superconductivity of Sr_2_RuO_4_ and certainly do not expect it to accurately predict *T*_c_(*ε*_100_) across the entire strain range, but it usefully highlights some of the key features of the experimental data. It demonstrates that the pronounced signal sign reversal that is so visually prominent in Fig. 3a for −0.35% < *ε*_100_ < −0.1% on entry to the superconducting state can be understood within a very simple model of superconductivity. Consistent with the trend seen in the data, the large entropy at the Van Hove singularity arising from the enhanced density of states is strongly quenched on entering the superconducting state. By contrast, a second model calculation shows that our data cannot be reproduced by superconducting states with nodes at the Van Hove points (see Supplementary Information). Our data are therefore consistent only with superconducting order parameters that give a substantial gap in the vicinity of the Van Hove singularity. Nodal lines or points away from the Van Hove point are, of course, still possible.

Arguably as interesting as what the model successfully describes is what it does not. The theory–experiment comparison in Fig. 3 again highlights the notable qualitative difference in the experimental data on the low-stain and high-strain sides of ${\varepsilon }_{100}^{{\rm{VHS}}}$. The models used to construct Figs. 2b and 3b do not include provision for a magnetic phase at high strain and predict a high degree of symmetry of both the normal state and the superconducting state signals around ${\varepsilon }_{100}^{{\rm{VHS}}}$. The pronounced asymmetry in the data shows that the magnetic state exists and suggests that it affects the superconductivity.

**Fig. 4:**
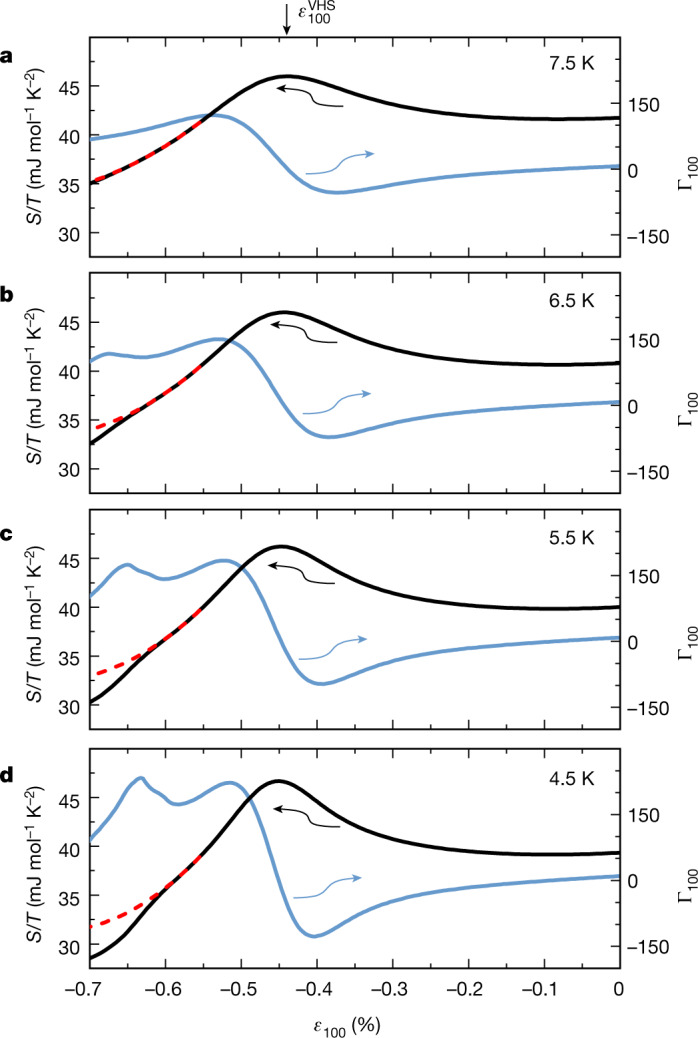
Strain dependence of the entropy. **a**–**d**, Integrating the Γ_100_ data using the iterative procedure described in Methods yields the strain-dependent entropy, plotted as *S*/*T* (black line) and directly compared with the measured Γ_100_ (blue line). The peak in Γ_100_ and corresponding rapid decrease of *S*/*T* indicates the magnetic transition at 4.5 K, 5.5 K and 6.5 K (**b**–**d**), whereas no decrease is visible at 7.5 K (**a**). The dotted red lines in the entropy traces are extrapolations of the background *S*/*T* from −0.55% to a lower cut-off consistent with the varying onset of the magnetic transition (−0.58%, −0.59%, −0.60% and −0.61% for 4.5 K, 5.5 K, 6.5 K and 7.5 K, respectively).

It is possible to go further than qualitative statements and to extract the strain dependence of the entropy, using the analysis procedure described in Methods. Sample results at 4.5 K, 5.5 K, 6.5 K and 7.5 K are shown in Fig. 4. After peaking at the Van Hove strain, the entropy decreases as the strain is increased, with a more sudden decrease for −0.61% < *ε*_100_ < −0.68%, whose magnitude increases with decreasing temperature. At a first-order phase transition, the entropy shows a discontinuity, whereas we observe instead a rapid decrease of finite width. However, our experiment involves a small strain inhomogeneity, whose effects are clearly seen in Fig. 3 in the broadening of the signal as the superconducting state is entered. The strain width of the entropy decrease in Fig. 4 is similar, so the data probably indicate that the intrinsic decrease is discontinuous. The raw elastocaloric data highlight the qualitative difference between the signature of a peaking entropy (seen at ${\varepsilon }_{100}^{{\rm{VHS}}}$) and the signature seen on entering the magnetic phase, which is a peak not in the entropy but in −(*∂S/∂ε*)_*T*_. Overall, although we cannot be absolutely certain, we believe that our data support a first-order transition into the magnetic phase. Taking the peak in Γ_100_ as the transition point identifies it with the dark blue ridge in Fig. 1e. We can also quantify the change of entropy. The decrease in *S*/*T* at 4.5 K is approximately 3 mJ mol^−1^ K^−2^, 8% of the electronic entropy of the unstrained material and more than 10% of the extrapolated background value at *ε*_100_ = −0.63%. The absolute value is similar to that seen on entry to the low-temperature phase in Sr_3_Ru_2_O_7_ (ref. ^11^) but the sign is opposite. In Sr_2_RuO_4_, the entropy is lower in the magnetic phase than in the adjacent metal, in line with conventional expectation for a Fermi surface gapping transition.

Independent of microscopic detail, the phase diagram determined by our measurements shows a strong and previously unappreciated experimental similarity between the tuned phase diagram of Sr_2_RuO_4_ and those of many cuprate, pnictide, organic and heavy fermion superconductors^25–28^, in which superconductivity appears in the vicinity of a magnetic phase that is driven towards zero temperature by an external tuning parameter. This is especially timely given the recent evidence for an even-parity order parameter in Sr_2_RuO_4_ (ref. ^9^), in common with those thought to exist in most of the above-mentioned materials. Our experiments also highlight the utility of the a.c. elastocaloric effect in the general study of unconventional superconductivity and correlated electron physics. As we have shown, the elastocaloric effect enables rapid and comprehensive phase diagram mapping and provides high-resolution datasets against which the quantitative predictions of theory can be tested. In the uniaxial pressure apparatus that we have developed^3,29^, its combination with other experiments should be fairly straightforward, offering simultaneous access to spectroscopic and thermodynamic information from the same sample.

## Methods

### Sample preparation and experimental setup

**Extended Data Fig. 4:**
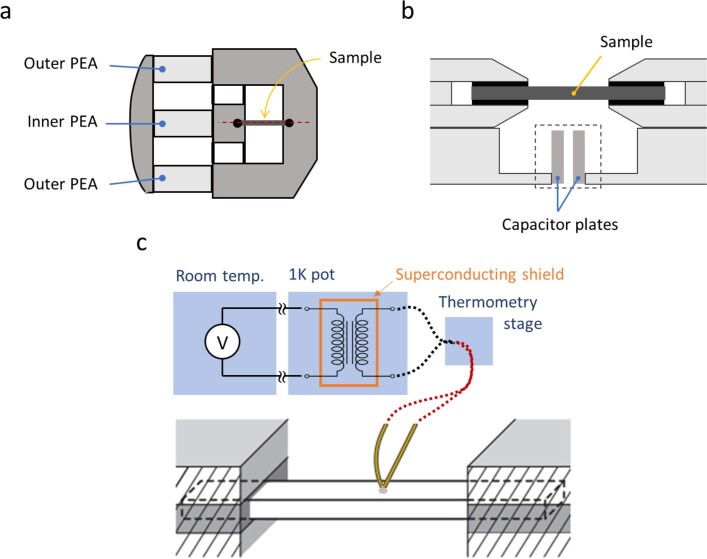
Schematic diagrams of the experimental setup. **a**, A uniaxial strain cell with inner and outer piezoelectric actuators (PEAs). To vary the tuning strain on the sample, the outer PEAs were driven between −350 V and 250 V. The inner PEA was kept constant at 185 V and the amplitude of the a.c. voltage to study the elastocaloric effect was between 0.25 V and 0.5 V. **b**, A detailed view of the mounted sample and the shielded capacitor plates, indicated by the dashed black box. The applied strain was determined by measuring the displacement of a capacitor mounted in parallel to the sample. **c**, The configuration of the electronic setup for the ultra-low-noise measurement of the temperature oscillations. The solid yellow lines represent the thermocouple, the dotted red lines the twisted copper wires and the dotted black lines twisted NbTi wires. The thermocouple wires are soldered to twisted copper wires, which were thermally anchored on the thermometry stage. From there, twisted superconducting wires, to reduce the input impedance, are connected to the input of a shielded low-temperature transformer, which is thermally anchored on the 1-K pot of the dilution refrigerator. Finally, the voltage is measured at room temperature using a lock-in amplifier.

High-quality single crystals of Sr_2_RuO_4_ were grown by a floating-zone method^31^. Special care was taken to select the sample from a region with the highest *T*_c_ and the absence of a signal from ‘3-K phase’ inclusions, indicating the highest quality Sr_2_RuO_4_. The sample was aligned along the [100] crystallographic direction by Laue X-ray diffraction and needles were wire sawed and polished using diamond-impregnated sheets with different grain sizes down to 1 μm to obtain parallel surfaces and to reduce the surface roughness. A home-made Au/AuFe (0.07%) thermocouple (25 μm wire diameter) served as a thermometer to measure the a.c. temperature changes. It was independently calibrated using procedures outlined in ref. ^30^ and attached to the centre of the sample using silver epoxy (Dupont 6838), soldered to twisted copper wires that were thermally anchored on the thermometry stage. The assembly was subsequently glued in the jaws of a uniaxial pressure cell using Stycast 2850FT epoxy with Catalyst 23LV. Special care was taken to minimize the tilt of the sample and to ensure a force transmission along the long axis of needle. The sample temperature was measured using calibrated resistive low-temperature sensors. The present experimental setup with the same sample was used in a previous heat capacity study^30,32^. Schematic diagrams of the experimental setup are shown in Extended Data Fig. 4a,b.

### Measurement of the elastocaloric effect

The elastocaloric effect was measured by an a.c. modulation method^18^. The uniaxial pressure apparatus was mounted to the cold plate of a dilution refrigerator (Oxford Instruments). To achieve the large strains needed to tune Sr_2_RuO_4_ in the desired range, large d.c. voltages had to be applied on both inner and outer piezoactuators of the uniaxial pressure apparatus. A home-made high-voltage amplifier was used to drive the outer piezoactuators. The a.c.-modulated strain was achieved by superimposing an a.c. voltage on top of a d.c. voltage on the inner piezoactuator. To amplify the coupled a.c. and d.c. voltages, a commercial high-voltage amplifier was used (TEGAM 2350, bandwidth d.c. to 2 MHz). The extremely low noise level of 20 pV (√Hz)^−1^ on the thermocouple readout, corresponding to 5.1 μK (√Hz)^−1^, 2.1 μK (√Hz)^−1^ and 1.7 μK (√Hz)^−1^ at 1 K, 4 K and 8 K, respectively, was obtained by the use of a high-frequency low-temperature transformer (CMR-Direct), operating at a gain of 300, mounted on the 1-K pot of the dilution refrigerator. Its output was read by an EG&G 7265 lock-in amplifier. We show the configuration of the electronic setup for the ultra-low-noise measurement of the temperature oscillations in Extended Data Fig. 4c.

### Determination of the applied uniaxial strain in the sample

Strain is the change of the length of a sample Δ*l* = *l* − *l*_0_ divided by its length *l*_0_. The strain apparatus used in this study has a capacitor to measure the displacement Δ*d* obtained by applying a voltage to the piezoelectric actuators (PEAs). However, the measured Δ*d* is not the change in the sample length. Δ*l* can be obtained by the change of the capacitor displacement Δ*d* times a transfer efficiency *e*, which is defined by the properties of Stycast layers between the sample and the jaws of the strain apparatus^3^. Therefore, we find for the strain in the sample:

3 $$\varepsilon =\frac{\Delta l}{l}=\frac{e\times \Delta d}{{l}_{0}}.$$

In the case of Sr_2_RuO_4_ in the current setup, a transfer efficiency *e* = 0.78 could be estimated on the basis of the known position of the maximum in *T*_c_ for an applied stress along [100] at 0.7 GPa (ref. ^29^) and the Young’s modulus *E*_Y_ = 160 GPa at 4 K (ref. ^33^).

To obtain the large strains needed to investigate the phase diagram of Sr_2_RuO_4_, the inner and outer PEAs of the strain apparatus are used. To measure the elastocaloric effect, a further small a.c. voltage is imposed on the d.c. voltage applied on the inner PEA. The oscillation amplitude *d*_exc_ can be measured using the capacitor mounted in parallel to the sample and the strain amplitude is then obtained following Equation (3):

4 $$\Delta \varepsilon =\frac{e\times {d}_{{\rm{exc}}}}{{l}_{0}}.$$

In our case, the displacement amplitude *d*_exc_ is between 5 nm and 10 nm, in comparison with a sample length of approximately 2 mm. Strain is a tensor quantity, so a formal definition of *ε*_100_ as used in the main text is $${\varepsilon }_{100}=$$ $${\vec{e}}_{100}$$$$\bullet \hat{\varepsilon }\bullet $$$${\vec{e}}_{100}$$, in which $${\vec{e}}_{100}=\left(\mathrm{1,0,0}\right)$$.

### Adiabaticity of the measurement

**Extended Data Fig. 5:**
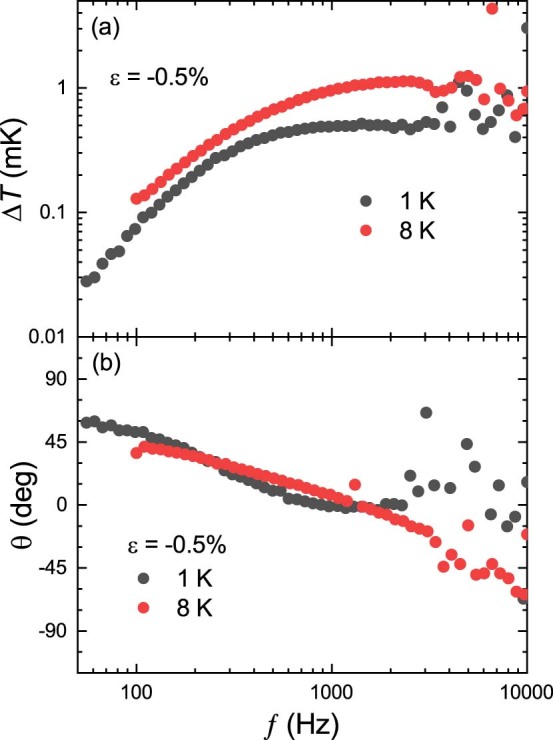
Frequency response of the thermocouple. Elastocaloric effect under 0.5% compression at 1 K and 8 K plotted against a logarithmic frequency scale. The applied strain oscillation is Δ*ε* = 3.45 × 10^−6^.

Curves of Δ*T* against frequency at 0.5% compression are shown in Extended Data Fig. 5 on a double-logarithmic representation. One can easily identify the lower cut-off frequency, between 100 Hz and 300 Hz. In the high-frequency range, this is not possible because the data start to scatter strongly above a few kilohertz before the upper cut-off frequency is reached. The enhanced noise is related to vibrations of thermocouple wires. Between 1 K and 8 K, we do not observe a notable change in the upper frequency boundary. This implies that the upper cut-off frequency is at least larger than 10 kHz. Here we chose a measuring frequency *f* = 1,513 Hz, which corresponds to Δ*T* on the plateau of the frequency response. The phase response is around zero for all temperatures between 1 K and 8 K at *f* = 1,513 Hz.

### Estimation of the elastocaloric signal size

In principle, the absolute value of the elastocaloric effect can be obtained directly. However, owing to the smallness of the signal and uncertainties arising from sample configuration and material properties, it is more reliable to calibrate the elastocaloric effect Δ*T*_ad_/Δ*ε*, as described in the following.

The elastocaloric effect can be described as an adiabatic temperature change Δ*T*_ad_ as a function of strain *ε*:

5 $$\frac{\Delta {T}_{{\rm{ad}}}}{\Delta \varepsilon }\cong -\frac{T}{{C}_{\varepsilon ,{\sigma }_{y},{\sigma }_{z}}}{\left(\frac{\partial S}{\partial \varepsilon }\right)}_{T,{\sigma }_{y},{\sigma }_{z}}$$

Here ${C}_{\varepsilon ,{\sigma }_{y},{\sigma }_{z}}$ is the heat capacity at constant strain and *S* is the entropy. The relevant elastocaloric Grüneisen parameter Γ in our experiment is related to entropy through

6 $$\Gamma =\frac{{\left(\partial S/\partial \varepsilon \right)}_{T,{\sigma }_{y},{\sigma }_{z}}}{{C}_{\varepsilon ,{\sigma }_{y},{\sigma }_{z}}}=\frac{{\left(\partial S/\partial \varepsilon \right)}_{T,{\sigma }_{y},{\sigma }_{z}}}{{T\left(\partial S/\partial T\right)}_{\varepsilon ,{\sigma }_{y},{\sigma }_{z}}}=-\frac{1}{T}{\left(\frac{\partial T}{\partial \varepsilon }\right)}_{S,{\sigma }_{y},{\sigma }_{z}}$$

Please note that, throughout this section, *ε* refers to *ε*_*xx*_. At very low strains on the order of −0.1% at temperatures above the superconducting transition, one can treat the system as being a Fermi liquid whose parameters are a function of strain. In this case, the specific heat to the second order in strain is given by

7 $$C\left(\varepsilon ,T\right)=\gamma \left(1+\varepsilon {\gamma }_{1}/\gamma +{{\varepsilon }^{2}\gamma }_{2}/\gamma \right)T+\beta {T}^{3}$$

Here we further assumed that the phonon heat capacity in our case has a negligible strain dependence. This is justified by both the small strain limit considered and the fact that the phonon contribution is much smaller than the electronic heat capacity at the relevant temperatures in the first place. It directly follows that entropy *S* is given by

8 $$S\left(\varepsilon ,T\right)={\int }_{0}^{T}\frac{C\left(\varepsilon ,T\right)}{T}{\rm{d}}T=\left(\gamma +{\gamma }_{1}\varepsilon +{\gamma }_{2}{\varepsilon }^{2}\right)T+\frac{1}{3}\beta {T}^{3}$$

In this limit, the elastocaloric Grüneisen parameter Γ can be expressed as

9 $$\Gamma =\frac{\left({\gamma }_{1}+{2\gamma }_{2}\varepsilon \right)T}{\left(\gamma +{\gamma }_{1}\varepsilon +{\gamma }_{2}{\varepsilon }^{2}\right)T+\beta {T}^{3}}$$

and

10 $${\left(\partial S/\partial \varepsilon \right)}_{T,{\sigma }_{y},{\sigma }_{z}}=\,\left({\gamma }_{1}+{2\gamma }_{2}\varepsilon \right)T$$

Furthermore, one can consider the second derivative of entropy with respect to strain

11 $${\left(\frac{{\partial }^{2}}{\partial {\varepsilon }^{2}}S\right)}_{T,{\sigma }_{y},{\sigma }_{z}}={\left(\frac{\partial }{\partial \varepsilon }{\left(\frac{\partial S}{\partial \varepsilon }\right)}_{T,{\sigma }_{y},{\sigma }_{z}}\right)}_{T,{\sigma }_{y},{\sigma }_{z}}=-{\left(\frac{\partial }{\partial \varepsilon }{\left(\frac{\partial {\sigma }_{x}}{\partial T}\right)}_{\varepsilon ,{\sigma }_{y},{\sigma }_{z}}\right)}_{T,{\sigma }_{y},{\sigma }_{z}}$$

in which we made use of the appropriate Maxwell relationship in the last step. Given that in the range considered here thermodynamic variables are well behaved, it follows that

12 $${\left(\frac{{\partial }^{2}}{\partial {\varepsilon }^{2}}S\right)}_{T,{\sigma }_{y},{\sigma }_{z}}=-{\left(\frac{\partial }{\partial T}{\left(\frac{\partial {\sigma }_{x}}{\partial \varepsilon }\right)}_{T,{\sigma }_{y},{\sigma }_{z}}\right)}_{\varepsilon ,{\sigma }_{y},{\sigma }_{z}}$$

in which stress *ε* and strain *σ* are related by means of the compliance matrix $\underline{\underline{{\bf{s}}}}$ through ${\boldsymbol{\varepsilon }}=\underline{\underline{{\bf{s}}}}{\boldsymbol{\sigma }}$. Hence

13 $${\left(\frac{{\partial }^{2}}{\partial {\varepsilon }^{2}}S\right)}_{T,{\sigma }_{y},{\sigma }_{z}}=-{\left(\frac{\partial }{\partial T}{s}_{11}^{-1}\right)}_{\varepsilon ,{\sigma }_{y},{\sigma }_{z}}$$

with *s*_11_ being the 11 entry of $\underline{\underline{{\bf{s}}}}$ and the inverse of the Young’s modulus.

Combining Equations (10) and (13) therefore yields

14 $$2{\gamma }_{2}T=-{\left(\frac{\partial }{\partial T}{s}_{11}^{-1}\right)}_{\varepsilon ,{\sigma }_{y},{\sigma }_{z}}$$

**Extended Data Fig. 6:**
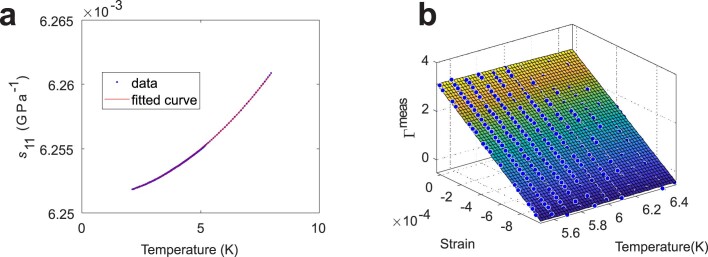
Data from a resonant ultrasound experiment and result of the calibration procedure of the Grüneisen parameter. **a**, *s*_11_ as a function of temperature determined in independent resonant ultrasound measurements together with a fit of the form shown in Equation (15). **b**, Measured Grüneisen parameter compared with the calibration function. The spheres correspond to the data and the surface to Equation (16) with the parameters mentioned in the text.

*s*_11_ determined from resonant ultrasound experiments using methods described in ref. ^33^ is shown in Extended Data Fig. 6a, together with a fit of the form

15 $${s}_{11}={s}_{11,0}+{s}_{11,2}{T}^{2}$$

giving *s*_11,2_ = 1.526 × 10^−7^ GPa^−1^ K^−2^ and ${\gamma }_{2}=\frac{{s}_{\mathrm{11,2}}}{{s}_{11}^{2}}\approx \frac{{s}_{\mathrm{11,2}}}{{s}_{\mathrm{11,0}}^{2}}=0.0039\,{\rm{GPa}}\,{{\rm{K}}}^{-2}$ .

The elastocaloric Grüneisen parameter is therefore fully determined except for *γ*_1_, permitting us to calibrate our measured data by means of an overall amplitude factor Γ = *a*Γ^meas^,

16 $${\Gamma }^{{\rm{meas}}}=\frac{1}{a}\,\frac{\left({\gamma }_{1}+2{\gamma }_{2}\varepsilon \right)T}{\left(\gamma +{\gamma }_{1}\varepsilon +{\gamma }_{2}{\varepsilon }^{2}\right)T+\beta {T}^{3}}$$

*γ*, *β* and *s*_11,2_ are constrained by independent experiments, with *a* and *γ*_1_ being the only independent parameters.

In Extended Data Fig. 6b, we show Γ^meas^ for temperatures between 5.5 K and 6.5 K and small strains for up to −0.1%, for which the above approximations are valid. The surface shown is a fit of the functional form of Equation (16). The fit gives *a* = 2.90 and *γ*_1_ = 6,797 J m^−3^ K^−1^.

### Numerical calculation of the entropy

Here we describe the numerical scheme for the calculation of entropy shown in Fig. 4.

Our starting point is

17 $${\Gamma }_{\varepsilon }=\frac{1}{{C}_{{\varepsilon }_{{xx}}}}{\left(\frac{\partial S}{\partial {\varepsilon }_{{xx}}}\right)}_{T}$$

Simple rearrangement gives

18 $${\left(\partial S/\partial {\varepsilon }_{{xx}}\right)}_{T}\,={C}_{{\varepsilon }_{{xx}}}{\Gamma }_{\varepsilon }$$

With the knowledge of the entropy at zero strain, *S*(*ε* = 0, *T*), one can integrate this partial differential to give

19 $$S\left(\varepsilon ,T\right)={\int }_{0}^{\varepsilon }{C}_{{\varepsilon }_{{xx}}}{\left(\varepsilon {\prime} ,T\right)\Gamma }_{\varepsilon }\left(\varepsilon {\prime} ,T\right){\rm{d}}\varepsilon {\prime} +S\left(\varepsilon =0,T\right)$$

Γ_*ε*_(*ε*, *T*) is known from the experiments, whereas ${C}_{{\varepsilon }_{xx}}(\varepsilon ,T)$ is, at this stage, unknown. However, it is related to *S*(*ε*, *T*) through

20 $${C}_{{\varepsilon }_{{xx}}}\left(\varepsilon {\prime} ,T\right)=T{\left(\frac{\partial S}{\partial T}\right)}_{{\varepsilon }_{{xx}}}$$

We therefore use an iterative scheme with the following steps. First, set ${C}_{{\varepsilon }_{xx}}^{(0)}({\varepsilon }_{xx},T)=C(0,T).$This is the zero-strain specific heat, known with very high accuracy for Sr_2_RuO_4_. Second, calculate ${S}^{\left(0\right)}\left({\varepsilon }_{{xx}},T\right)$ using Equation (19) and ${C}_{{\varepsilon }_{{xx}}}^{\left(0\right)}\left({\varepsilon }_{{xx}},T\right)$ for all available *T*. Third, calculate ${C}_{{\varepsilon }_{xx}}({\varepsilon }_{xx},T)$ by interpolating ${S}^{\left(0\right)}\left({\varepsilon }_{{xx}},T\right)$ as a function of *T* and evaluating Equation (20). Finally, calculate ${S}^{\left(1\right)}\left({\varepsilon }_{{xx}},T\right)$ using ${C}_{{\varepsilon }_{{xx}}}^{\left(1\right)}\left({\varepsilon }_{{xx}},T\right)$ and Equation (19), an iteration of the second step.

After a few iterations, no notable changes in *S*^(*n*)^(*ε*, *T*) are observed. This is not least due to the fact that, although ${C}_{{\varepsilon }_{{xx}}}$ does vary overall, these variations are at most a few tens of per cent, enabling an effective convergence of the above scheme. The data shown in the main text correspond to *S*^(2)^.

### Theoretical analysis

The theoretical analysis of the elastocaloric effect is on the basis of a quasiparticle description of Sr_2_RuO_4_. We use a strain-dependent quasiparticle dispersion *ε*_**k**_(*ϵ*_*αβ*_) and determine the electronic contribution to the entropy of the system from

$${S}_{{\rm{el}}}=-\frac{2{k}_{{\rm{B}}}}{N}\,{\sum }_{{\bf{k}}}\left[\,{f}_{{\bf{k}}}{\rm{\log }}\,{f}_{{\bf{k}}}+\left(1-{f}_{{\bf{k}}}\,\right){\rm{\log }}\left(1-{f}_{{\boldsymbol{k}}}\,\right)\right].$$

*f*_**k**_ is the Fermi distribution function with the above dispersion. The factor 2 refers to the electron spin and the sum goes over the momenta in the first Brillouin zone. The elastocaloric coefficient follows from the temperature and strain derivatives of the entropy. We use the following tight-binding parameterization for the *γ*-band of the system as determined from angle-resolved photoemission experiments^34^:

$${\varepsilon }_{{\bf{k}}}=-2{t}_{x}{\rm{\cos }}({k}_{x}{a}_{x})-2{t}_{y}{\rm{\cos }}({k}_{y}{a}_{y})-4t{\prime} {\rm{\cos }}({k}_{x}{a}_{x}){\rm{\cos }}({k}_{y}{a}_{y})-\mu ,$$

with *t*_*x*_ = *t*_*y*_ = *t*_0_ = 0.119 eV, *t*′ = 0.392*t*_0_ and *μ* = 1.48*t*_0_. To describe the strain dependence of *ε*_**k**_(*ϵ*_*αβ*_), we assume a linear dependence of the hopping elements with respect to the interatomic distance. The proportionality factor is chosen to reproduce the strain value at which the Van Hove singularity is reached. In the superconducting state, we use the Bogoliubov quasiparticle dispersion ${\varepsilon }_{{\bf{k}}}\to \sqrt{{\varepsilon }_{{\bf{k}}}^{2}+{\Delta }^{2}}$ with superconducting gap Δ. The strain dependence is dominated by the electronic spectrum near the Van Hove point. In our theory, we consider a pairing state that is fully gapped at the Van Hove momentum. The strain dependence of the superconducting gap amplitude and of the transition temperature follow from the solution of the gap equation at fixed pairing interaction. For details, see the Supplementary Information.

## Online content

Any methods, additional references, Nature Research reporting summaries, source data, extended data, supplementary information, acknowledgements, peer review information; details of author contributions and competing interests; and statements of data and code availability are available at 10.1038/s41586-022-04820-z.

## Supplementary information

Supplementary Methods This file contains details about the theoretical considerations and about the a.c. strain amplitude. It includes more figures showing the results of calculations.

## Figures and Tables

**Extended Data Fig. 1:**
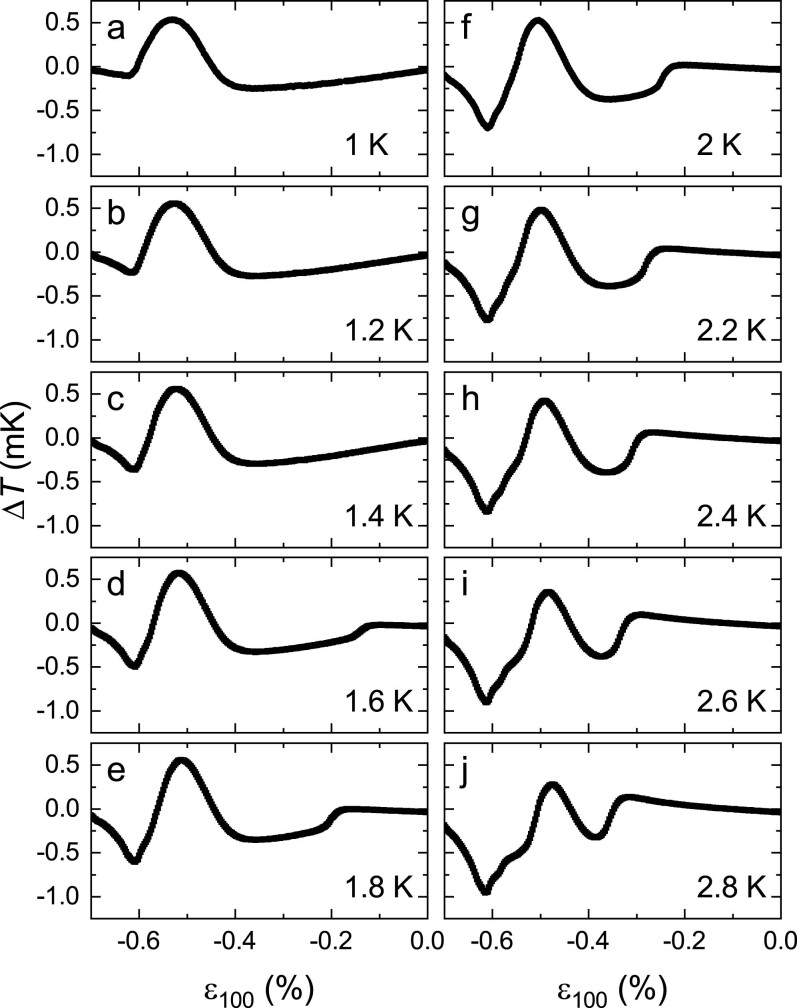
Response of the elastocaloric effect as a function of strain (further data: low temperatures). Δ*T*(*ε*_100_) recorded at 1,513 Hz with an excitation amplitude ${\varepsilon }_{100}^{{\rm{exc}}}$ between 2.9 × 10^−6^ and 3.5 × 10^−6^ during strain sweeps at ten different temperatures from 1 K to 2.8 K.

**Extended Data Fig. 2:**
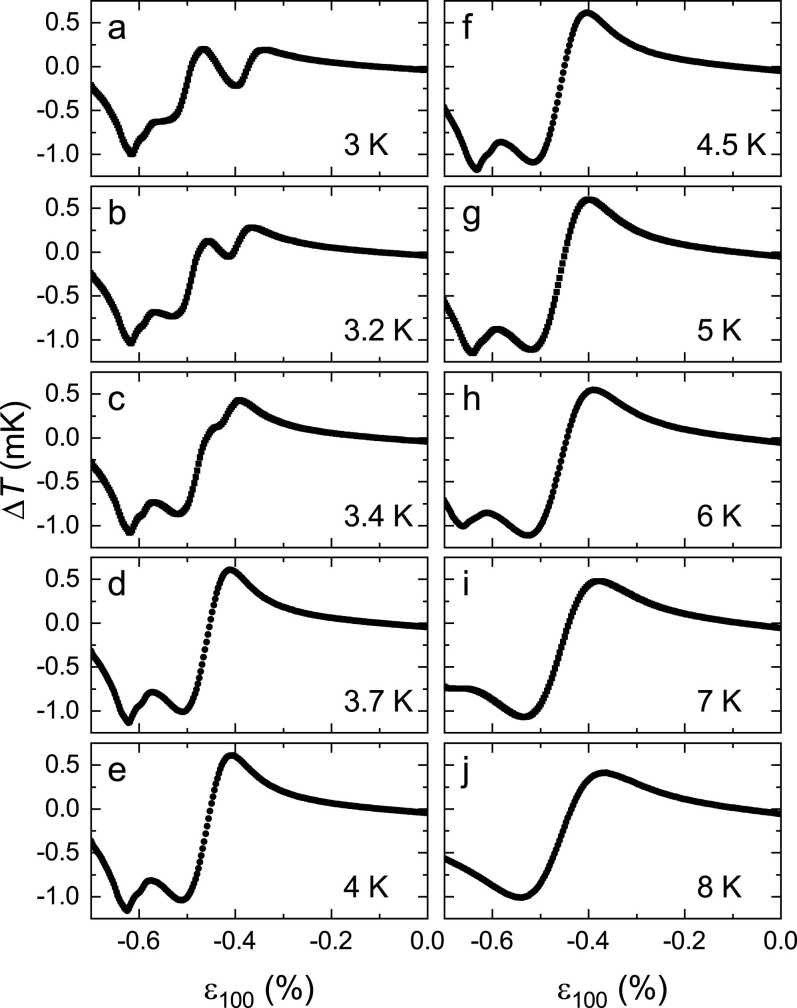
Response of the elastocaloric effect as a function of strain (further data: high temperatures). Δ*T*(*ε*_100_) recorded at 1,513 Hz with an excitation amplitude ${\varepsilon }_{100}^{{\rm{exc}}}$ between 2.9 × 10^−6^ and 3.5 × 10^−6^ during strain sweeps at ten different temperatures from 3 K to 8 K.

**Extended Data Fig. 3:**
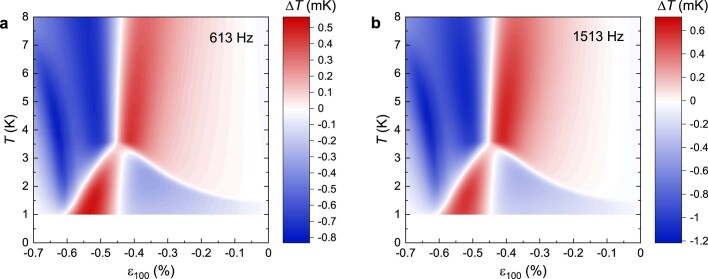
Comparison of the colour maps of the elastocaloric effect taken at different frequencies. Data taken at 613 Hz (**a**) and at 1,513 Hz (**b**). The data were recorded with an excitation amplitude ${\varepsilon }_{100}^{{\rm{exc}}}$ between 2.9 × 10^−6^ and 3.5 × 10^−6^.

## Data Availability

The data that underpin the findings of this study are available at 10.17630/6a4a06c6-38d3-464f-88d1-df8d2dbf1e75.
