# Supplementary material for: Elastocaloric determination of the phase diagram of Sr2RuO4
Source: Nature. 2022 Jul 13;607(7918):276–80. doi: 10.1038/s41586-022-04820-z (PMC9279151; doi:10.1038/s41586-022-04820-z)
Supplement: Supplementary file 1 — This file contains details about the theoretical considerations and about the a.c. strain amplitude. It includes more figures showing the results of calculations. [file 41586_2022_4820_MOESM1_ESM.pdf]

nature portfolio

https://doi.org/10.1038/s41586-022-04820-z

Supplementary information

Elastocaloric determination of the phase diagram of Sr_2RuO_4

In the format provided by the authors and unedited

Elastocaloric determination of the phase diagram of Sr_2RuO_4

You-Sheng Li,[1] Markus Garst,[2,3] Jörg Schmalian,[3,4] Sayak Ghosh,[5] Naoki Kikugawa,[6] Dmitry A. Sokolov,[1] Clifford W. Hicks,[1,7] Fabian Jerzembeck,[1] Matthias S. Ikeda,[8,9,10] Zhenhai Hu,[1] B. J. Ramshaw,[5] Andreas W. Rost,[11,12] Michael Nicklas,[1] and Andrew P. Mackenzie[1,11]

[1] *Max Planck Institute for Chemical Physics of Solids, Nöthnitzer Str. 40, 01187 Dresden, Germany*

[2] *Institut für Theoretische Festkörperphysik, Karlsruher Institut für Technologie, 76131 Karlsruhe, Germany*

[3] *Institut für Quantenmaterialien und -technologien, Karlsruher Institut für Technologie, 76131 Karlsruhe, Germany*

[4] *Institut für Theorie der Kondensierten Materie, Karlsruher Institut für Technologie, 76131 Karlsruhe, Germany*

[5] *Laboratory of Atomic and Solid State Physics, Cornell University, Ithaca, NY, USA*

[6] *National Institute for Materials Science, Tsukuba 305-0003, Japan*

[7] *School of Physics and Astronomy, University of Birmingham, Birmingham B15 2TT, United Kingdom*

[8] *Geballe Laboratory for Advanced Materials, Stanford University, 476 Lomita Mall, Stanford, California 94305, USA*

[9] *Department of Applied Physics, Stanford University, 348 Via Pueblo Mall, Stanford, California 94305, USA*

[10] *Stanford Institute for Materials and Energy Science, SLAC National Accelerator Laboratory, 2575 Sand Hill Road, Menlo Park, California 94025, USA*

[11] *Scottish Universities Physics Alliance, School of Physics and Astronomy, University of St Andrews, St Andrews, UK*

[12] *Max Planck Institute for Solid State Research, Heisenbergstr. 1, 70569 Stuttgart, Germany*

I. FREQUENCY, TEMPERATURE, AND STRAIN DEPENDENCE OF THE AC STRAIN AMPLITUDE

In our study of the elastocaloric effect we used the *in situ* determined displacement amplitude d_{exc} to calculate the applied AC strain amplitude $\Delta\varepsilon$. In the following we demonstrate that the applied displacement amplitude d_{exc} displays only small variations as function of frequency, temperature, and strain in the parameter range of our measurements.

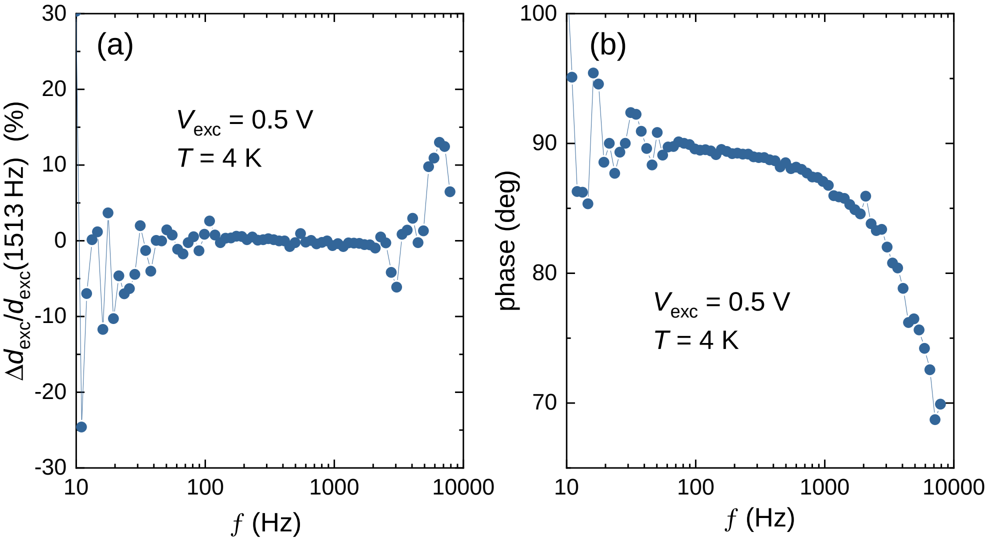

Figure S1. Frequency sweeps of (a) d_{exc} and the corresponding (b) phase shift at zero applied tuning strain.

Figure S1 displays the frequency response of the AC displacement amplitude and the corresponding phase shift at 4 K for an excitation amplitude of 0.5 V. d_{exc} is in a wide range independent of frequency. Below about 70 Hz we observe an increased scattering of the data. At high-frequency, above 2 kHz, we find an abrupt increase in $d_{exc}(f)$ indicating a nonlinear voltage-displacement response of the piezo-electric actuator. In this frequency range also the phase shift starts to deviate considerably from 90°. We conclude that in our set-up, the response of the actuators is appropriate for measurement in the range between 70 Hz and approximately 2 kHz.

The AC displacement amplitude exhibits a small temperature dependence. It monotonically increases by approximately 6% between 1 and 8 K. Figure S2 shows data taken at a frequency of 1513 Hz with an AC voltage amplitude V_{exc} = 0.5 V used to drive the piezo-electric actuator.

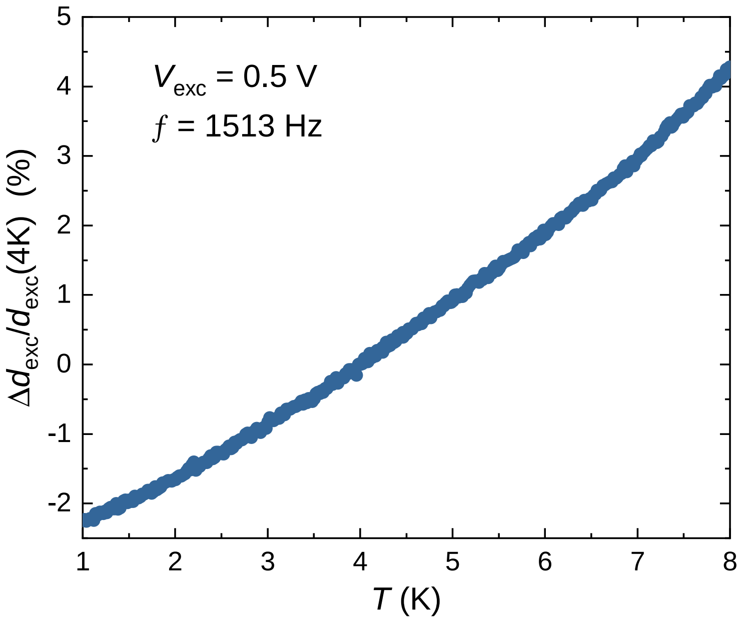

Figure S2. Temperature sweeps of $\Delta d_{exc} = [d_{exc} - d_{exc}(4\mathrm{K})]/d_{exc}(4\mathrm{K})$ at zero applied tuning strain.

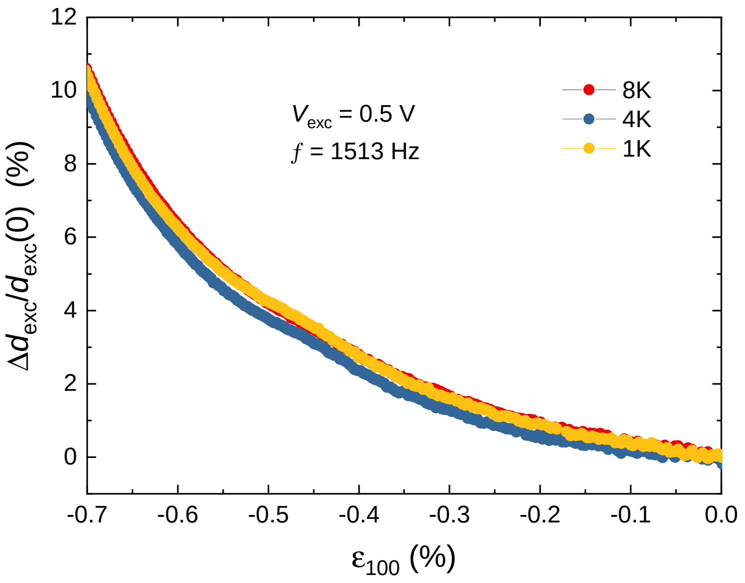

Figure S3. Tuning strain sweeps of $\Delta d_{exc} = [d_{exc} - d_{exc}(\varepsilon_{100} = 0)]/d_{exc}(\varepsilon_{100} = 0)$ at different temperatures.

The same frequency and AC voltage amplitude was used to investigate the dependence of d_{exc} on applied tuning strain. Figure S3 displays data at three selected temperatures. The general behavior of $d_{exc}(\varepsilon)$ does not change with temperature. At all temperature the

maximum change in $d_{exc}(\varepsilon)$ is less than 11 % over the range of strain used in our experiments.

II. THEORETICAL CONSIDERATIONS

In section II A we provide a review on the relation between the elastocaloric effect and the uniaxial Grüneisen parameter Γ. In section II B we provide details of the calculation of Γ across the superconducting transition close to the strain-induced Van-Hove singularity of Sr_2RuO_4 using a parametrisation of its γ-band; some results of this calculation are shown in the main text. Finally, in section II C we discuss the entropy quench across the superconducting transition within an effective theory valid close to the Van-Hove singularity, and we compare the scenario of a full gap versus a gap with nodes at the Van-Hove point in momentum space. We find that a nodal gap at the Van-Hove point is inconsistent with experimental signatures.

A. Elastocaloric effect and uniaxial Grüneisen parameter

In the case that the temperature T and uniaxial strain, say, along the x-axis ε_{xx} can be controlled experimentally the entropy should be considered as a function $S = S(T, \varepsilon_{xx})$. Its differential is then given by

$$dS(T, \varepsilon_{xx}) = \frac{\partial S}{\partial T}\bigg|_{\varepsilon_{xx}} dT + \frac{\partial S}{\partial \varepsilon_{xx}}\bigg|_T d\varepsilon_{xx} = \frac{C_{\varepsilon_{xx}}}{T} dT + \frac{\partial S}{\partial \varepsilon_{xx}}\bigg|_T d\varepsilon_{xx}, \tag{S1}$$

where $C_{\varepsilon_{xx}} = T \frac{\partial S}{\partial T}\Big|_{\varepsilon_{xx}}$ is the specific heat at constant uniaxial strain ε_{xx}. Under adiabatic conditions $dS = 0$, it follows

$$\Gamma = -\frac{1}{T} \frac{dT}{d\varepsilon_{xx}}\bigg|_S = \frac{\frac{\partial S}{\partial \varepsilon_{xx}}\Big|_T}{C_{\varepsilon_{xx}}}, \tag{S2}$$

where Γ is the Grüneisen parameter generalized to the case of uniaxial strain ε_{xx}. Up to a factor of T, it quantifies the change of temperature upon adiabatically varying ε_{xx} and thus describes an elastocaloric effect. The Grüneisen parameter defined in Eq. S2 is dimensionless.

The derivative $\frac{\partial S}{\partial \varepsilon_{xx}}\Big|_T$ can be related to the uniaxial thermal expansion, $\alpha_{xx} = \frac{\partial \varepsilon_{xx}}{\partial T}\Big|_{\sigma_{zz}} = \frac{\partial S}{\partial \sigma_{xx}}\Big|_T$, and Young's modulus, $\frac{1}{E_Y} = \frac{\partial \sigma_{xx}}{\partial \varepsilon_{xx}}\Big|_T$,

$$\frac{\partial S}{\partial \varepsilon_{xx}}\bigg|_T = \frac{\partial S}{\partial \sigma_{xx}}\bigg|_T \frac{\partial \sigma_{xx}}{\partial \varepsilon_{xx}}\bigg|_T = \frac{\alpha_{xx}}{E_Y}, \tag{S3}$$

where σ_{xx} is the corresponding stress component. The uniaxial Grüneisen parameter is then given by $\Gamma = \alpha_{xx}/(C_{\varepsilon_{xx}} E_Y)$.

B. Grüneisen parameter arising from the γ-band of Sr_2RuO_4

1. *Tight-binding model for the γ-band*

We follow Refs.[1, 2] and consider an experimentally determined tight-binding model of the γ-band of Sr_2RuO_4 with the two-dimensional electron spectrum

$$\varepsilon_{\boldsymbol{k}} = -2t_x \cos(k_x a_x) - 2t_y \cos(k_y a_y) - 4t' \cos(k_x a_x) \cos(k_y a_y) - \mu \tag{S4}$$

where μ is the chemical potential. The nearest-neighbour hopping amplitudes are t_x and t_y, and t' is the next-nearest-neighbour hopping amplitude within the crystallographic (a, b)-plane with lattice constants a_x and a_y.

In the absence of strain the system possesses tetragonal symmetry with lattice constants $a_x = a_y$. In order to obtain the values for the hopping parameters at zero strain, we make use of tight-binding fits to the ARPES data by Burganov *et al.* [3] that yields the values $t_x = t_y = t_0 = 0.119$ eV, $t'_0 = 0.392 t_0$, and $\mu = 1.48 t_0$. The value for t_0 and the chemical potential μ are directly taken from Ref. [3]. For the next-nearest neighbour hopping Burganov *et al.* give the value $t'_0 = 0.41 t_0$; however, this value yields an energy of 19 meV at the point $(k_x, k_y) = (0, \pi)$ in the two-dimensional Brillouin zone that overestimates the value obtained experimentally. Instead of a global fit performed by Burganov *et al.* [3], we refitted their data with a local fit optimizing the behavior around this point in the Brillouin zone yielding a value for t'_0 that is 4% smaller. We note that the value for t'_0 of Burganov *et al.* [3] will not qualitatively change the behavior of the Grüneisen parameter but change its magnitude by approximately a factor of two due to an overestimate of the electronic energy.

In the presence of normal strains ε_{xx} and ε_{yy}, the amplitudes are modified, and for small strains we can approximate up to linear order

$$t_x = t_0(1 - \alpha\varepsilon_{xx}), \quad t_y = t_0(1 - \alpha\varepsilon_{yy}), \quad t' = t'_0(1 - \alpha'(\varepsilon_{xx} + \varepsilon_{yy})/2), \tag{S5}$$

with linear coefficients α and α'. The uniaxial strain along the y-direction, $\varepsilon_{yy} = -\nu_{xy}\varepsilon_{xx}$, is determined by ε_{xx} with the Poisson ratio whose value at ambient pressure and 4 K is

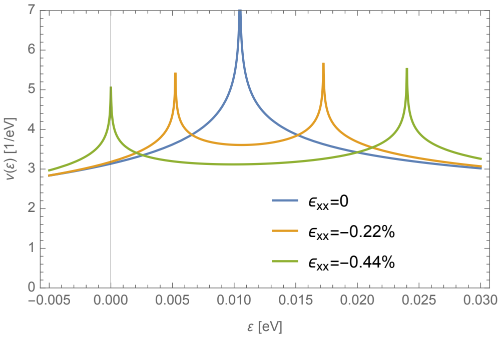

Figure S4. Density of states S6 associated with the γ-band of Sr_2RuO_4 in units of 1/eV as a function of energy for various values of uniaxial strain ε_{xx}. A single Van-Hove singularity for zero strain splits into two for finite ε_{xx}. A Van-Hove singularity reaches the Fermi level at $\varepsilon = 0$ for $\varepsilon_{xx} = -0.44\%$ (green line).

given by $\nu_{xy} \approx 0.508$ [4]. The Van-Hove singularity is reached in the experiment for a strain of $\varepsilon_{xx} = -0.44\%$, see above. The tight-binding dispersion exhibits a Van-Hove point at $(k_x, k_y) = (0, \pi)$ for a choice of the parameters $\alpha = \alpha' \approx 7.604$, where we used $\alpha = \alpha'$ for simplicity. This value is consistent with the calculations of Barber *et al.* [5]. The parameter α will determine the overall magnitude of the Grüneisen parameter.

The corresponding density of states per spin is given by

$$\nu(\varepsilon) = a_x a_y \int_{-\pi/a_x}^{\pi/a_x} \frac{dk_x}{2\pi} \int_{-\pi/a_y}^{\pi/a_y} \frac{dk_y}{2\pi} \delta(\varepsilon - \varepsilon_{\boldsymbol{k}}). \tag{S6}$$

We have multiplied the integral by the area of the two-dimensional unit cell, $a_x a_y$, so that $\nu(\varepsilon)$ represent the number of states per energy and per unit cell. This density of states is independent of the lattice constants, as becomes manifest upon substituting the integration variables $k_x = \tilde{k}_x / a_x$ and $k_y = \tilde{k}_y / a_y$. The strain dependence of $\nu(\varepsilon)$ thus arises from the hopping amplitudes in the dispersion of Eq. S4. $\nu(\epsilon)$ and other densities of states that we use in the analysis of the Grüneisen parameter can be expressed in terms of elliptic integrals.

The evolution of the energy-dependent density of states with uniaxial strain is illustrated in Fig. S4. The Van-Hove singularity at zero uniaxial strain splits into two for finite ε_{xx}. A Van-Hove singularity reaches the Fermi level for $\varepsilon_{xx} = -0.44\%$. The density of states at the Fermi level $\nu(0)$ as a function of uniaxial strain is shown in Fig. S5. A Van-Hove singularity at the Fermi level is realized for compressive strain $\varepsilon_{xx} \approx -0.44\%$ as well as tensile strain $\varepsilon_{xx} \approx 0.34\%$. In between the density of states is minimal for $\varepsilon_{xx} \approx -0.07\%$.

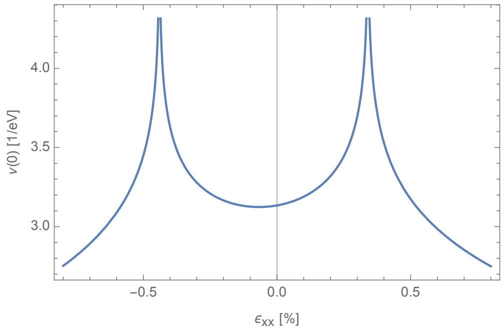

Figure S5. Density of states S6 at the Fermi level $\nu(0)$ as a function of uniaxial strain ε_{xx}. A Van-Hove singularity at the Fermi level is realized for compressive strain $\varepsilon_{xx} \approx -0.44\%$ as well as tensile strain $\varepsilon_{xx} \approx 0.34\%$. As a result, a minimum arises at $\varepsilon_{xx} \approx -0.07\%$.

2. *Thermodynamics arising from the γ-band*

In order to account for the behavior of the Grüneisen parameter across the superconducting transition close to the Van-Hove singularity of Sr_2RuO_4, we consider the entropy per unit cell,

$$S(T, \varepsilon_{xx}) = -2k_B \int d\varepsilon\, \nu(\varepsilon)\Big(f(E)\log f(E) + (1 - f(E))\log(1 - f(E))\Big) \tag{S7}$$

where $E(\varepsilon) = \sqrt{\varepsilon^2 + \Delta^2}$ with the superconducting gap Δ, and the Fermi function $f(E) = (e^{E/(k_B T)} + 1)^{-1}$. The factor of 2 in front of the integral of Eq. S7 accounts for the spin degree of freedom. The superconducting gap Δ is determined by the gap equation

$$\frac{1}{g} = \int_{-\hbar\omega_D}^{\hbar\omega_0} d\varepsilon\, \nu(\varepsilon) \frac{\tanh \frac{E}{2k_B T}}{2E} \tag{S8}$$

with a cutoff provided by the frequency ω_0, and the coupling constant $g > 0$ with units of energy. Our choice for the cutoff frequency $\hbar\omega_0/k_B = 400$ K was informed by inelastic neutron scattering data on Sr_2RuO_4 [6]. In order to reproduce the critical temperature $T_c \approx 3.5$ K as observed at the strain-induced Van-Hove singularity of Sr_2RuO_4, we need to choose $g/k_B \approx 714$ K. The gap $\Delta = \Delta(T, \varepsilon_{xx})$ itself will not only depend on temperature T but also on strain ε_{xx}, see Figure S6.

With the help of the entropy S7 the Grüneisen parameter can be evaluated using Eq. S2 by taking derivatives with respect to T and strain ε_{xx}. The specific heat is given by

$$C_{\varepsilon_{xx}} = T\frac{\partial S}{\partial T}\Big|_{\varepsilon_{xx}} = \frac{2k_B}{4(k_B T)^2} \int d\varepsilon\, \nu(\varepsilon) \frac{E^2 - T\Delta\partial_T\Delta}{\cosh^2(E/(2k_B T))}. \tag{S9}$$

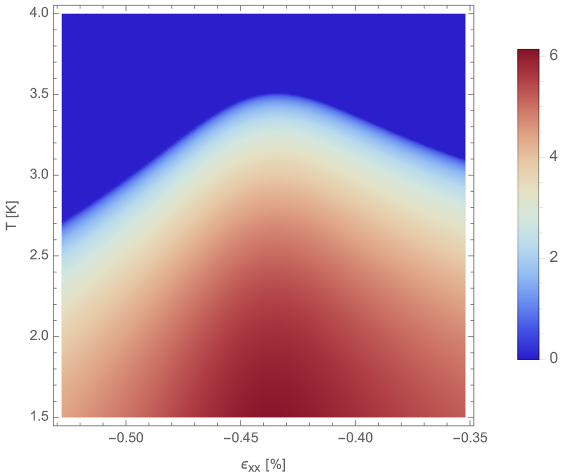

Figure S6. Superconducting gap Δ/k_B evaluated with Eq. S8 in units of Kelvin as a density plot within the (ε_{xx}, T) plane. The maximal critical temperature $T_c|_{\max} \approx 3.5$ K is obtained close to the Van-Hove singularity $\varepsilon_{xx} \approx -0.44\%$.

For the strain derivative we obtain

$$\frac{\partial S}{\partial \varepsilon_{xx}}\Big|_T = \frac{2k_B}{4(k_B T)^2} \int d\varepsilon \, \frac{\nu_{\varepsilon_{xx}}(\varepsilon)\varepsilon - \nu(\varepsilon)\Delta\partial_{\varepsilon_{xx}}\Delta}{\cosh^2(E/(2k_B T))}, \tag{S10}$$

where we introduced the auxiliary function

$$\nu_{\varepsilon_{xx}}(\varepsilon) = a_x a_y \int_{-\pi/a_x}^{\pi/a_x} \frac{dk_x}{2\pi} \int_{-\pi/a_y}^{\pi/a_y} \frac{dk_y}{2\pi} \frac{\partial \varepsilon_{\boldsymbol{k}}}{\partial \varepsilon_{xx}} \delta(\varepsilon - \varepsilon_{\boldsymbol{k}}). \tag{S11}$$

In the normal phase $\Delta = 0$ sufficiently far away from the Van Hove singularity where the density of states $\nu(\varepsilon)$ only smoothly varies in the range $|\varepsilon| \lesssim k_B T$ around the Fermi energy, standard Fermi liquid behaviour is obtained. The entropy then depends linearly on temperature

$$S \approx \frac{2\pi^2}{3} k_B^2 T \nu(0), \tag{S12}$$

and the Grüneisen parameter reduces to a constant

$$\Gamma \approx \frac{1}{\nu(0)} \frac{\partial \nu(0)}{\partial \varepsilon_{xx}}, \tag{S13}$$

that quantifies the strain dependence of the density of states at the Fermi level $\nu(0)$. From this expression follows that the Grüneisen parameter in the Fermi-liquid limit will change sign at maxima as well as minima of $\nu(0)$ as a function of uniaxial strain ε_{xx}. The pronounced

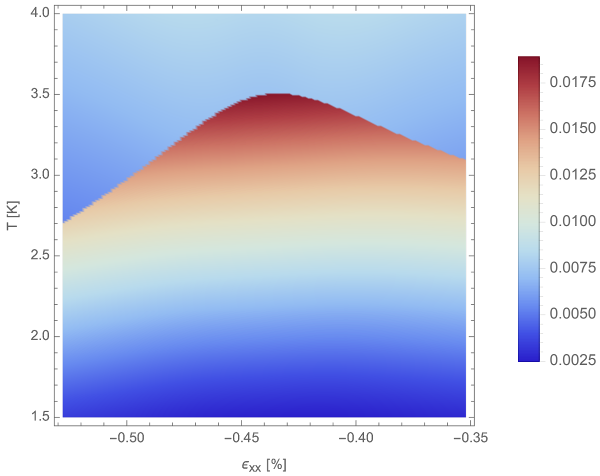

Figure S7. Specific heat $C_{\varepsilon_{xx}}$ evaluated with Eq. S9 in units of k_B per unit cell as a density plot within the (ε_{xx}, T) plane.

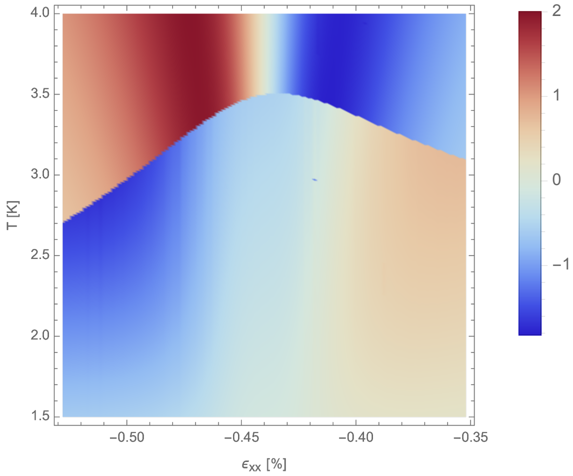

Figure S8. Strain derivative of the entropy $\partial S/\partial \varepsilon_{xx}|_T$ evaluated with Eq. S10 in units of k_B per unit cell as a density plot within the (ε_{xx}, T) plane.

sign change of Γ close to the Van Hove singularity $\varepsilon_{xx} = -0.44\%$ where $\nu(0)$ is maximal is clearly observed in the experimental data, see figure 3 in the main text. The additional sign change in the experimental data close to zero strain is in agreement with the expected minimum of $\nu(0)$, see Fig. S5.

The evaluation of the specific heat, the strain derivative of entropy and the Grüneisen parameter are shown as a function of temperature T and uniaxial strain ε_{xx} in Figs. S7, S8 and S9, respectively. Additional line cuts are shown in the main text. At the Van Hove

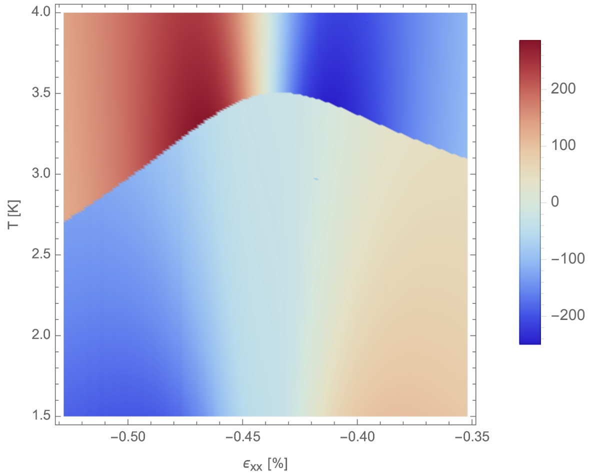

Figure S9. Grüneisen parameter $\Gamma = (\partial S/\partial\varepsilon_{xx}|_T)/C_{\varepsilon_{xx}}$ using the results of Figs. S7 and S8 as a density plot within the (ε_{xx}, T) plane.

singularity above the critical temperature, entropy accumulates resulting in a maximum of $S(\varepsilon_{xx})$ and thus a sign change of $\partial S/\partial\varepsilon_{xx}|_T$ and Γ. As the superconducting phase is entered upon lowering the temperature, a large part of the entropy is quenched so that the maximum in $S(\varepsilon_{xx})$ is converted into a minimum. This leads to a saddle point in the strain dependence of the entropy $S(\varepsilon_{xx})$ close to the maximum of the critical temperature T_c.

3. Influence of inter-layer hopping on the Van Hove singularity

A finite inter-plane hopping of electrons will eventually cut off the logarithmic divergence of the Van Hove singularity in the density of states and, as a consequence, weaken and broaden the associated thermodynamic signatures. Here, we estimate the temperature and strain scale at which this cut-off is expected to happen.

We start the discussion with an extended tight-binding Hamiltonian following Ref. [7] that is based on the three t_{2g} Ru orbitals,

$$\mathcal{H}_s(\vec{k}) = \begin{pmatrix} \varepsilon_{AA}(\vec{k}) & \varepsilon_{AB}(\vec{k}) - is\eta & \varepsilon_{AC}(\vec{k}) + i\eta \\ \varepsilon_{AB}(\vec{k}) + is\eta & \varepsilon_{BB}(\vec{k}) & \varepsilon_{BC}(\vec{k}) - s\eta \\ \varepsilon_{AC}(\vec{k}) - i\eta & \varepsilon_{BC}(\vec{k}) - s\eta & \varepsilon_{CC}(\vec{k}) \end{pmatrix}. \tag{S14}$$

Here, $s = \pm 1$ represents the spin degree of freedom, η is the spin-orbit coupling, and the

dispersions are parameterised as

$$\begin{aligned}\varepsilon_{AA}(k_x,k_y,k_z) =& -2t_1\cos(k_x)-2t_2\cos(k_y)-2t_3\cos(2k_x)-4t_4\cos(k_x)\cos(k_y) \\ &-4t_5\cos(2k_x)\cos(k_y)-2t_6\cos(3k_x)-2t_7\cos(2k_y) \\ &-2t_8\cos(k_x/2)\cos(k_y/2)\cos(k_z)-\mu_{1D},\end{aligned} \tag{S15}$$

$$\varepsilon_{BB}(k_x,k_y,k_z) = \varepsilon_{AA}(k_y,k_x,k_z), \tag{S16}$$

$$\begin{aligned}\varepsilon_{CC}(k_x,k_y,k_z) =& -2\bar{t}_1(1-\alpha\varepsilon_{xx})\cos(k_x)-2\bar{t}_1(1+\alpha\nu_{xy}\varepsilon_{xx})\cos(k_y) \\ &-4\bar{t}_2(1-\frac{\alpha'}{2}(1-\nu_{xy})\varepsilon_{xx})\cos(k_x)\cos(k_y)-2\bar{t}_3(\cos(2k_x)+\cos(2k_y)) \\ &-4\bar{t}_4(\cos(2k_x)\cos(k_y)+\cos(2k_y)\cos(k_x)) \\ &-\bar{t}_5\cos(k_x/2)\cos(k_y/2)\cos(k_z)-\mu_{2D},\end{aligned} \tag{S17}$$

$$\varepsilon_{AB}(k_x,k_y,k_z) = -4t_{\text{int},1}\sin(k_x)\sin(k_y)-4t_{\text{int},2}\sin(k_x/2)\sin(k_y/2)\cos(k_z), \tag{S18}$$

$$\varepsilon_{AC}(k_x,k_y,k_z) = -4t_{\text{int},3}\cos(k_x/2)\sin(k_y/2)\sin(k_z), \tag{S19}$$

$$\varepsilon_{BC}(k_x,k_y,k_z) = -4t_{\text{int},3}\cos(k_y/2)\sin(k_x/2)\sin(k_z). \tag{S20}$$

We used already dimensionless wavevectors, $a_ik_i \to k_i$ with $i = x, y, z$ and $k_i \in (-\pi, \pi]$. This Hamiltonian gives rise to the α, β, and γ bands. The values of the hopping parameters can be found in Ref. [7] where they were obtained by a fit to *ab initio* calculations. We already accounted for a strain dependence ε_{xx} of the hopping parameters $\bar{t}_1$ and $\bar{t}_2$ with a strength α and α', similar to Eq. S5, and neglected for simplicity the dependence of all other hopping parameters on strain.

Note that the *ab initio* parameters of Ref. [7] overestimate the values of the hopping parameters. For example, the value $\bar{t}_1 = 0.3568$ eV [7] should be compared to $t_0 = 0.119$ eV of section II B 1. Using the *ab initio* hopping parameters of Ref. [7] and the Poisson ratio $\nu_{xy} = 0.508$ [4] setting $\alpha' = \alpha$ for simplicity, the γ band dispersion $\varepsilon_\gamma(\vec{k})$ of Eq. S14 vanishes at $(k_x, k_y, k_z) = (0, \pi, 0)$ for the critical strain $\varepsilon_{xx}^{\text{VH}} = -0.44\%$ for a value of the parameter $\alpha = 15.62$. These values based on *ab initio* calculations will be used for the following estimates.

First, let us recall the behavior of the γ band of the two-dimensional model of section II B 1. Close to the point $(k_x, k_y) = (0, \pi)$ its dispersion possesses a saddle point

$$\varepsilon_\gamma(k_x,k_y,0) \approx c_1k_x^2 - c_2(k_y-\pi)^2 + c_3\delta\varepsilon_{xx}, \tag{S21}$$

with positive coefficients c_1, c_2, c_3, and $\delta\varepsilon_{xx} = \varepsilon_{xx} - \varepsilon_{xx}^{\rm VH}$ is the distance to the critical strain. This leads to a logarithmic singularity in the two-dimensional density of states

$$\nu_{2d}(\varepsilon) = \int_{-\pi}^{\pi} \frac{dk_x}{2\pi} \int_{-\pi}^{\pi} \frac{dk_y}{2\pi} \delta(\varepsilon - \varepsilon_\gamma(k_x, k_y, 0)) \sim \frac{1}{8\pi^2\sqrt{c_1 c_2}} \log \frac{1}{|\varepsilon - c_3\delta\varepsilon_{xx}|}. \tag{S22}$$

The dispersion of the γ band resulting from the full three-dimensional model of Eq. S14 possesses close to $(k_x, k_y, 0) = (0, \pi, k_z)$ the modified form

$$\varepsilon_\gamma(\vec{k}) \approx c_1 k_x^2 - c_2(k_y - \pi)^2 + c_3\delta\varepsilon_{xx} + c_4(k_y - \pi)\cos(k_z) - c_5 k_x(k_y - \pi)\sin(k_z) + c_6\sin^2(k_z). \tag{S23}$$

We kept here only the first order Fourier components of k_z except for the last term with coefficient c_6 that involves a second order $2k_z$ Fourier component. We also neglected contributions of order $\mathcal{O}(k_x^2\delta\varepsilon)$ and $\mathcal{O}((k_y - \pi)^2\delta\varepsilon)$. Using the values of the hopping parameters of Ref. [7] we obtain for the coefficients the following estimates $c_2 \approx 0.395$ eV, $c_1/c_2 \approx 0.15$, $c_4/c_2, c_5/c_2 \sim 0.01$, $c_6 \approx 0.0005$ eV and $c_3 \approx 12.950$ eV. The contribution c_4 and c_5 shift the position of the saddle point and can effectively be absorbed into small shift of c_1, c_2 and c_6 by substitution of the wavevectors (k_x, k_y). The main effect arises from the coefficient c_6 that effectively leads to a k_z-dependence of the tuning parameter $\delta\varepsilon_{xx}$. It cuts off the logarithmic singularity in the three dimensional density of states,

$$\nu_{3d}(\varepsilon) \sim \frac{1}{8\pi^2\sqrt{c_1 c_2}} \int_{-\pi}^{\pi} \frac{dk_z}{2\pi} \log \frac{1}{|\varepsilon - c_3\delta\varepsilon_{xx} - c_6\sin^2(k_z)|}. \tag{S24}$$

The resulting density of states for $\delta\varepsilon_{xx} = 0$ is approximately constant in the range $|\varepsilon| < c_6$ with two cusps, i.e., typical non-analyticities for a three-dimensional density of states at the two edges $\varepsilon = \pm c_6$, see Fig. S10(a).

The regularization of the logarithmic singularity in the density of states by the inter-layer hopping leads to a temperature scale $T_{3d} \sim c_6/k_B$ and a strain scale $\varepsilon_{xx}^{3d} \sim c_6/c_3$. Using the above estimates we obtain the values $T_{3d} \approx 5.8$ K and $\delta\varepsilon_{xx}^{3d} \sim 3.8 \times 10^{-5}$, i.e., approximately 10^{-2} of the absolute value of the critical strain. The latter is consistent with comparing T_{3d} and the energy of the γ band at $(k_x, k_y, k_z) = (0, \pi, 0)$ and zero strain that is 53 meV or 615 K using the *ab initio* parameters (compared to just over 10 meV measured experimentally). Both scales are reflected for example in the dependence of the specific heat coefficient of the normal state. In the low temperature limit, on the one hand, the specific heat coefficient is proportional to the density of states. In order to resolve the inter-layer regularization

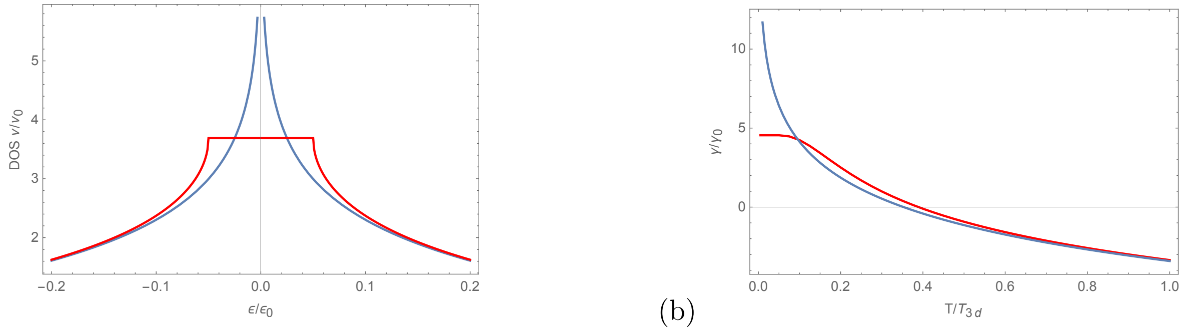

(a) (b)

Figure S10. (a) Regularization of the two-dimensional Van Hove singularity in the density of states $\nu(\varepsilon) = \nu_0 \log(\varepsilon/\varepsilon_0)$ (blue line) at the critical strain $\delta\varepsilon_{xx} = 0$ via the inter-plane hopping that cuts off the singularity giving rise to two cusps (red line). For this illustration the coefficient was chosen to be $c_6 = 0.1\varepsilon_0$. (b) Specific heat coefficient $\gamma = C/T$ for the density of states of panel (a) (up to a constant background) as a function of temperature with $\gamma_0 = \nu_0 k_B$ where k_B is the Boltzmann constant. The two-dimensional logarithmic divergence (blue line) is cut off by the inter-layer hopping and the specific heat coefficient saturates below a temperature of order $0.1T_{3d}$.

a strain resolution of order $\delta\varepsilon_{xx}^{3d}$ would be required that is far beyond the experimental resolution. On the other hand, at the critical strain the specific heat coefficient diverges logarithmically as a function of decreasing temperature for the two-dimensional model. For the three dimensional model with inter-layer hopping this divergence is cut off, and the specific heat coefficient saturates but only below a temperature of order $0.1T_{3d} \sim 0.6$ K, see Fig. S10(b).

We can conclude for Sr_2RuO_4 that its thermodynamics in the normal state is only barely affected by the inter-layer hopping. First, its signatures as a function of strain occur on a scale beyond the strain resolution of the experiment. Second, its signatures as a function of temperature are most pronounced at the critical strain but are negligible for temperatures $T > T_{c,\max} \approx 3.5$ K $\approx 0.6T_{3d}$. For lower temperatures the Van Hove singularity seems to be rather cut off by the superconducting order parameter masking the effect of the inter-layer hopping on the Van Hove singularity in density of states. Finally, note that the *ab initio* parameters overestimate the scales so that our estimates about the influence of inter-layer hopping are on the conservative side.

4. Influence of disorder on the Van Hove singularity

The Van Hove singularity might in principle also be smeared out by elastic scattering off impurities. This becomes relevant once the elastic scattering rate $\hbar\tau_0^{-1}$ exceeds the scale for inter-layer coupling, which corresponds to several Kelvin [8]. We are, however, confident that the scattering rate must be significantly below this value. Sr_2RuO_4 is known to be sensitive to pair-breaking due to non-magnetic impurities [9]. Hence, the very observation of a superconducting transition implies that $\hbar\tau_0^{-1} \ll k_\mathrm{B}T_\mathrm{c}$ and elastic scattering events will not smear out the Van Hove singularity in the temperature regime of our measurements.

C. Entropy quench for a superconducting gap with nodes at the Van Hove singularity

In section II B it was assumed that the superconductor is fully gapped at the Van Hove point in momentum space. In this case, the calculated entropy, that is enhanced at the Van Hove singularity, gets strongly quenched upon entering the superconducting phase, see Fig. S9. This behavior is consistent with the experimental observations as presented in the main text. In the present section, we demonstrate that a superconducting gap with nodes at the Van Hove points is not able to reproduce such a substantial entropy quench.

1. Effective low-energy theory for the Van Hove singularity and nodal superconductivity

For this purpose, we consider an effective theory that is only valid close to the Van Hove singularity. Expanding the dispersion of Eq. S4 close to the Van Hove singularity at $k_y = \pi/a_y$, it acquires in lowest order the following form

$$\varepsilon_{\boldsymbol{q}} = \frac{1}{2m}(q_x^2 - q_y^2) + r \tag{S25}$$

where $r = -\mu + 2(t_y - t_x) + 4t'$ tunes the distance to the Van Hove point, the mass $m = \frac{1}{2a_x a_y\sqrt{(t_x-2t')(t_y+2t')}}$ and the wavevectors are $q_y = (k_y - \frac{\pi}{a_y})\sqrt{b}$ and $q_x = k_x/\sqrt{b}$ with the dimensionless scaling factor $b = \frac{a_y}{a_x}\sqrt{\frac{t_y+2t'}{t_x-2t'}}$. We will supplement the effective theory with a hard energy cutoff $(q_x^2 + q_y^2)/(2m) \leq \varepsilon_0$ where ε_0 is on the order of the hopping; we will use $\varepsilon_0 = \frac{1}{2ma_xa_y}$.

We assume that the superconducting gap depends in general analytically on momentum, $\Delta_{\boldsymbol{q}} = \Delta\gamma_{\boldsymbol{q}}$, and we consider the following three scenarios:

$$\gamma_{\boldsymbol{q}} = \begin{cases} 1 & \text{scenario (1)} \\ a_x a_y (q_x^2 - q_y^2) & \text{scenario (2)} \\ a_x a_y 2 q_x q_y & \text{scenario (3).} \end{cases} \tag{S26}$$

The scenario (1) corresponds to a hard gap at the Van Hove singularity, and this should reproduce the asymptotic behavior of section II B. The scenarios (2) and (3) correspond to a nodal superconductor. Whereas for case (2) the location of the nodes coincides with the location of the Fermi surface at $r = 0$, the scenario (3) still produces a gap for a generic point on the Fermi surface for $r = 0$ that decreases, however, and vanishes with distance to the location of the Van Hove point $|\boldsymbol{q}| = 0$ in momentum space.

The superconductor possesses the dispersion $E_{\boldsymbol{q}} = \sqrt{\varepsilon_{\boldsymbol{q}}^2 + \Delta_{\boldsymbol{q}}^2}$. For a momentum-dependent gap, it is convenient to consider in this section the density of states defined as follows

$$\nu(E) = a_x a_y \int_{\varepsilon_0} \frac{dq_x dq_y}{(2\pi)^2} \delta(E - \sqrt{\varepsilon_{\boldsymbol{q}}^2 + \Delta_{\boldsymbol{q}}^2}), \tag{S27}$$

where the integral over wavevectors is evaluated with the hard energy cutoff $\varepsilon_0 \geq (q_x^2 + q_y^2)/(2m)$. It is clear that $\nu(E)$ is symmetric in r because the case $r < 0$ can be mapped onto $r > 0$ by interchanging q_x and q_y in the definition of $\nu(E)$. In the following, we therefore focus on positive $r > 0$.

The density of states is illustrated in Fig. S11 for the three gap scenarios of Eq. S26. The fully gapped scenario (1) produces a hard gap in the density of states $E_{\text{gap}} = \Delta$. The nodal scenario (2) also produces a small hard gap whose size however depends on both the tuning parameter and the cutoff, $E_{\text{gap}} = \frac{\Delta|r|}{\sqrt{\varepsilon_0^2+\Delta^2}}$; right at the Van Hove point $r = 0$, the singularity in $\nu(E)$ is maintained. The nodal scenario (3) caps the Van Hove singularity in the density of states at $r = 0$ and gives rise to a plateau with constant $\nu(E)$ up to energies $E \leq \Delta$; for any finite $r \neq 0$ a soft gap appears.

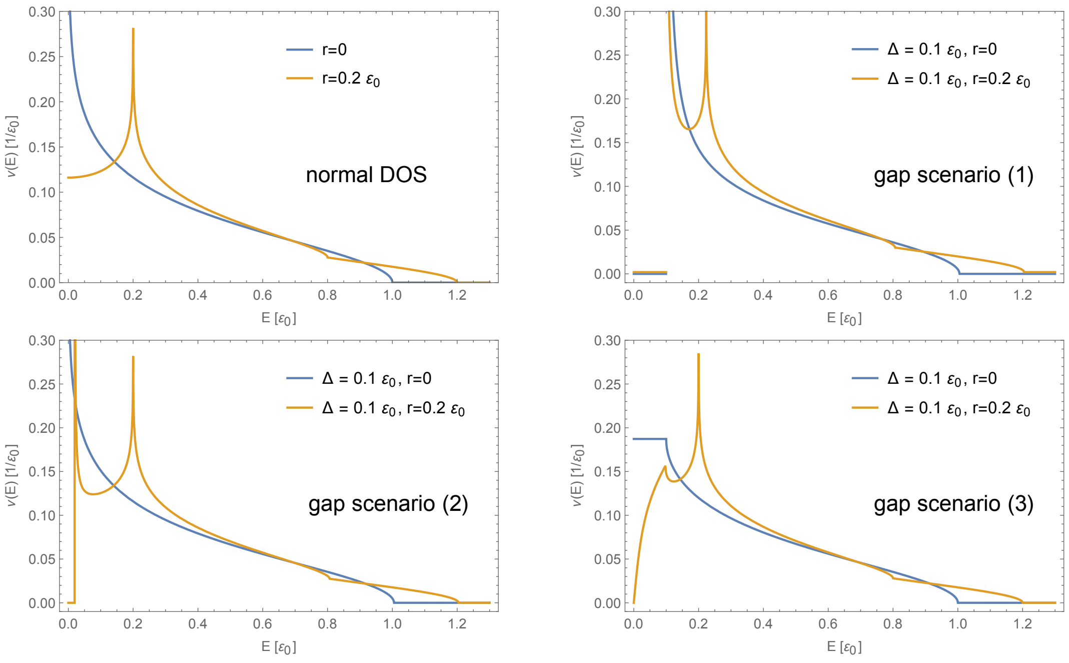

Figure S11. Density of states S27 for various values of the tuning parameter r and the gap Δ. The different panels illustrate the three scenarios for the superconducting gap, see Eq. S26.

2. *Gap equation and phase diagram*

The gap Δ fulfils the gap equation

$$\frac{1}{g} = \int_0^{E_0} dE\, \nu_\gamma(E) \frac{\tanh \frac{E}{2k_B T}}{2E}, \tag{S28}$$

with the coupling constant g, another cutoff $E_0 \ll \varepsilon_0$, and the auxiliary density of states is given by

$$\nu_\gamma(E) = a_x a_y \int_{\varepsilon_0} \frac{dq_x dq_y}{(2\pi)^2} \gamma_{\boldsymbol{q}}^2\, \delta(E - \sqrt{\varepsilon_{\boldsymbol{q}}^2 + \Delta_{\boldsymbol{q}}^2}). \tag{S29}$$

It turns out that the auxiliary density of states $\nu_\gamma(E)$ for $\Delta = 0$ in the gap scenario (2) vanishes at the Van Hove point $r = 0$ as $\nu_\gamma(E) \propto E^2 \log 1/E$ for $E \to 0$. At zero energy, $E = 0$, and finite r it assumes a finite value $\nu_\gamma(0) \propto r^2 \log 1/|r|$ for small r. As a result, the critical temperature $T_c(r)$ possesses a minimum at $r = 0$ and increases for increasing distance to the Van Hove point. As this behavior is inconsistent with the experimental signatures, we exclude this scenario (2) in the following.

For the numerical evaluation, we choose for the cutoffs $\varepsilon_0 = 0.1$ eV and $E_0 = 0.01$ eV. Moreover, we demand that the critical temperature $T_{c,\max} = 3.5$ K, i.e., $k_B T_{c,\max}/\varepsilon_0 \approx 0.003$

at the Van Hove singularity, $r = 0$, in order to connect with experiment. The coupling constant g is chosen accordingly. The resulting phase diagrams for the scenarios (1) and (3) in the (r, T) plane are shown in Fig. S12.

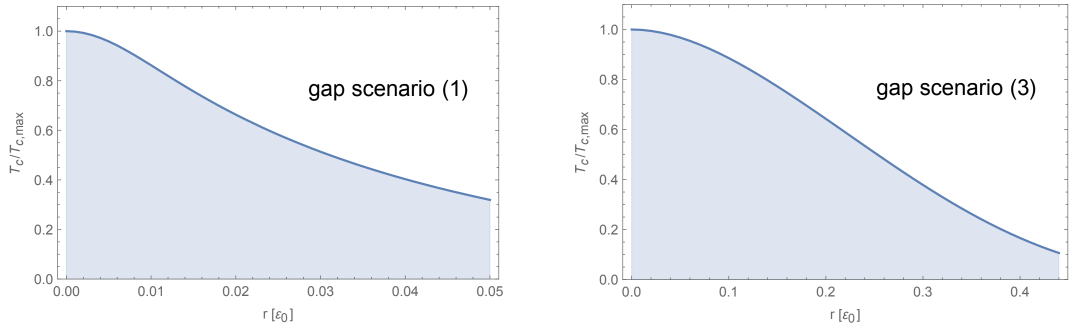

Figure S12. Phase diagrams in the (r, T) plane for the gap scenarios (1) and (3) with a common $T_{c,\max} = 3.5$ K. Note the different scales for the tuning parameter r on the x-axis. The gap scenario (2) possesses a critical temperature $T_c(r)$ that is minimal at $r = 0$ and, as a consequence, it is not considered further.

3. *Entropy quench across the superconducting transition*

Here, we discuss the behavior of entropy close to the top of both superconducting domes in Fig. S12. The entropy per unit cell and per spin is given by

$$S(T, r) = -2k_B \int_0^\infty dE\, \nu(E)\Big(f(E)\log f(E) + (1 - f(E))\log(1 - f(E))\Big). \tag{S30}$$

It is symmetric $S(T, r) = S(T, -r)$ and we focus thus on positive $r \geq 0$. The entropy as a function of r is evaluated for a few temperatures in Fig. S13.

The panels show the entropy divided by temperature because in the Fermi liquid limit this ratio is proportional to the density of states of the normal state, see also Eq. S12. This limiting behavior is expected for $T > T_c(r)$ and $r \gg k_B T$. As $k_B T_{c,\max} \approx 0.003\,\varepsilon_0$ this amounts to $r/\varepsilon_0 \gg 0.003$ for $T \approx T_{c,\max}$. Due to the different scales for the tuning parameter on the x-axis, this limit is mostly fulfilled in Fig. S13 for scenario (3) (provided that $T > T_c(r)$) but not yet for scenario (1).

As the superconducting dome is entered as a function of decreasing r, the entropy for the fully gapped scenario (1) exhibits a characteristic kink where the entropy changes slope

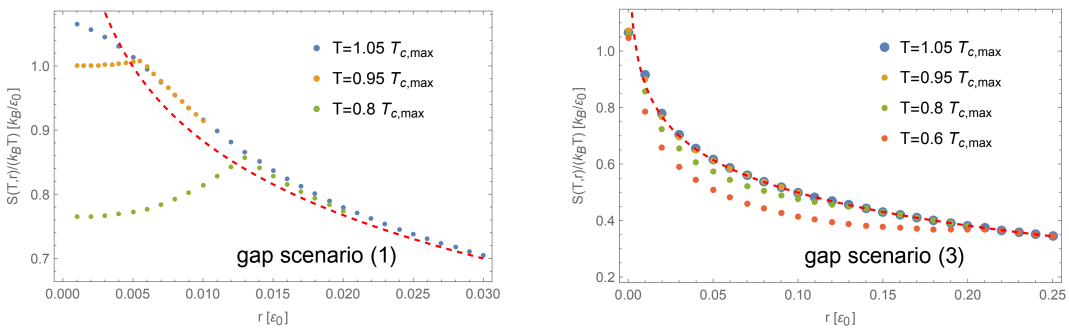

Figure S13. Entropy divided by temperature close to the top of the superconducting dome for scenarios (1) and (3) as a function of the tuning parameter r. The red dashed line corresponds to $S = \frac{\pi^2}{3} k_B^2 T \nu(0)|_{\Delta=0}$ and is proportional to the density of states in the normal state, see also the discussion in the context of Eq. S12.

$\partial_r S$ even close to the top of the dome for $T = 0.95\, T_{c,\text{max}}$. As a consequence, the maximum in $S(r)$ at $r = 0$ for $T > T_{c,\text{max}}$ is rapidly converted into a minimum for $T < T_{c,\text{max}}$. The behaviour for the nodal gap scenario (3) is distinctly different. As its density of states at the Van Hove point $r = 0$ is only capped by superconductivity but remains gapless, see Fig. S11, the entropy remains basically unquenched at $r = 0$ upon entering the superconducting dome. A small quench only appears for finite r at $T < T_c(r)$ when the density of states develops a soft gap. This small quench is however not able to change the sign of the slope $\partial_r S$ at least not down to temperatures of $T = 0.6\, T_{c,\text{max}}$.

Close to the Van Hove singularity, $r = 0$, the strain sensitivity is mostly attributed to the strain dependence of the tuning parameter, $r = r(\varepsilon_{xx})$. As a consequence, the slope $\partial_r S(r)$ is basically proportional to the strain derivative $\partial_{\varepsilon_{xx}} S$. A sign change of $\partial_r S$ is thus directly reflected in a sign change of the elastocaloric effect. The behavior for the fully gapped scenario (1) is, as expected, consistent with the calculation of section II B. It is also consistent with the experimentally observed signatures whereas the nodal gap scenario is not. So we conclude that the conversion of the maximum in entropy $S(\varepsilon_{xx})$ into a minimum upon entering the superconducting dome at its top as derived from measurements of the elastocaloric effect on Sr_2RuO_4 strongly suggests a superconducting order with a full gap at

the Van Hove point in momentum space.

---

[1] C. Bergemann, A. P. Mackenzie, S. R. Julian, D. Forsythe, and E. Ohmichi, Quasi-two-dimensional fermi liquid properties of the unconventional superconductor Sr_2RuO_4, Adv. Phys. **52**, 639 (2003).

[2] M. E. Barber, A. S. Gibbs, Y. Maeno, A. P. Mackenzie, and C. W. Hicks, Resistivity in the vicinity of a van Hove singularity: Sr_2RuO_4 under uniaxial pressure, Phys. Rev. Lett. **120**, 076602 (2018).

[3] B. Burganov, C. Adamo, A. Mulder, M. Uchida, P. D. C. King, J. W. Harter, D. E. Shai, A. S. Gibbs, A. P. Mackenzie, R. Uecker, M. Bruetzam, M. R. Beasley, C. J. Fennie, D. G. Schlom, and K. M. Shen, Strain control of fermiology and many-body interactions in two-dimensional ruthenates, Phys. Rev. Lett. **116**, 197003 (2016).

[4] S. Ghosh, A. Shekhter, F. Jerzembeck, N. Kikugawa, D. A. Sokolov, M. Brando, A. P. Mackenzie, C. W. Hicks, and B. J. Ramshaw, Thermodynamic evidence for a two-component superconducting order parameter in Sr_2RuO_4, Nat. Phys. **17**, 99 (2021).

[5] M. E. Barber, F. Lechermann, S. V. Streltsov, S. L. Skornyakov, S. Ghosh, B. J. Ramshaw, N. Kikugawa, D. A. Sokolov, A. P. Mackenzie, C. W. Hicks, and I. I. Mazin, Role of correlations in determining the van Hove strain in Sr_2RuO_4, Phys. Rev. B **100**, 245139 (2019).

[6] M. Braden, Y. Sidis, P. P. Bourges, P., J. Kulda, Z. Mao, and M. Y., Inelastic neutron scattering study of magnetic excitations in Sr_2RuO_4, Phys. Rev. B **66**, 064522 (2002).

[7] H. S. Røising, T. Scaffidi, F. Flicker, G. F. Lange, and S. H. Simon, Superconducting order of Sr_2RuO_4 from a three-dimensional microscopic model, Phys. Rev. Res. **1**, 033108 (2019).

[8] A. P. Mackenzie and Y. Maeno, The superconductivity of Sr_2RuO_4 and the physics of spin-triplet pairing, Rev. Mod. Phys. **75**, 657 (2003).

[9] A. P. Mackenzie, R. K. W. Haselwimmer, A. W. Tyler, G. G. Lonzarich, Y. Mori, S. Nishizaki, and Y. Maeno, Extremely strong dependence of superconductivity on disorder in Sr_2RuO_4, Phys. Rev. Lett. **80**, 161 (1998).
